# Evaluation of the Anticancer Activity of Medicinal Plants Predominantly Accumulating Ellagic Acid Compounds

**DOI:** 10.3390/antiox14111339

**Published:** 2025-11-06

**Authors:** Domantas Armonavičius, Audrius Maruška, Baltramiejus Jakštys, Mantas Stankevičius, Tomas Drevinskas, Kristina Bimbiraitė-Survilienė, Modesta Čaplikaitė, Hirotaka Ihara, Makoto Takafuji, Elżbieta Skrzydlewska, Ona Ragažinskienė, Yutaka Kuwahara, Shoji Nagaoka, Vilma Kaškonienė, Saulius Šatkauskas, Arvydas Kanopka

**Affiliations:** 1 Instrumental Analysis Open Access Centre, Institute of Research of Natural and Technological Sciences, Faculty of Natural Sciences, Vytautas Magnus University, Vileikos St. 8, LT-44404 Kaunas, Lithuania; 2 Research on Delivery of Medicine and Genes Cluster, Institute of Research of Natural and Technological Sciences, Faculty of Natural Sciences, Vytautas Magnus University, LT-44001 Kaunas, Lithuania; baltramiejus.jakstys@vdu.lt (B.J.);; 3 Faculty of Advanced Science and Technology, Kumamoto University, Kumamoto 860-8555, Japantakafuji@kumamoto-u.ac.jp (M.T.);; 4 Department of Analytical Chemistry, Medical University of Białystok, 15-222 Białystok, Poland; 5 Botanical Garden, Vytautas Magnus University, LT-46324 Kaunas, Lithuania; 6 Kumamoto Industrial Research Institute, Kumamoto University, Kumamoto 860-8555, Japan

**Keywords:** antioxidants, phenolic compounds, ellagitannins, oenothein B, HPLC-ED, anticancer, cytotoxicity

## Abstract

Cancer remains a major global health challenge, prompting the search for natural therapeutic agents with selective anticancer activity. This study investigated extracts from 12 medicinal plant species (a total of 21 samples) rich in phenolic compounds, particularly ellagic acid and its derivatives, to evaluate their antioxidant properties and ability to inhibit cancer cell viability. Spectrometric analysis and high-performance liquid chromatography (HPLC) with electrochemical detection (ED) and ultraviolet–visible (UV-VIS) detection were used for compound identification. The anticancer activity of plant extracts was tested using the MTS cell proliferation assay to determine anticancer activity on 4T1, A549, Caki-1, HCT116, and MCF7 cancer cell lines. The HEK-293 healthy cell line was used to determine extracts cytotoxicity. Study results indicate that black walnut (*Juglans nigra* L.), fireweed (*Chamaenerion angustifolium* L.), and pedunculate oak (*Quercus robur* L.) have the highest contents of bioactive compounds. Among tested extracts, fireweed showed the lowest IC_50_ values, thus the strongest anticancer activity against 4T1 cells (IC_50_ = 0.28 ± 0.01 RE (rutin equivalents) mg/g), while black walnut was most effective against Caki-1 and HCT116 (IC_50_ = 1.56 ± 0.01; 2.56 ± 0.02 RE mg/g). IC_50_ values are reported in rutin equivalents (RE) to maintain consistency with antioxidant normalization parameters used throughout the study. Extended incubation increased anticancer activity across most medicinal plant extracts, with fireweed and Canadian goldenrod demonstrating rapid and sustained potency already at 24 h. After 72 h, the most active fireweed extract inhibited approximately 104–190 million cancer cells per gram of dried plant material, demonstrating substantial antiproliferative activity consistent with the IC_50_ findings. Importantly, none of the extracts showed cytotoxicity to healthy HEK-293 cells. Overall, the findings highlight several plant species with significant anticancer potential, underscoring their promise as sources of natural bioactive compounds for future cancer prevention and treatment research.

## 1. Introduction

Cancer constitutes a significant public health concern, exerting a considerable influence on millions of individuals worldwide. According to the 2020 World Health Organization (WHO) and International Agency for Research on Cancer (IARC), an estimated 20 million new cancer cases and 9.7 million deaths occurred in 2022, with the number of new cases projected to reach over 35 million by 2050, representing a 77% increase from 2022 (WHO, GLOBOCAN, 2024 [1]). Despite recent progress in cancer therapies [2,3], the pursuit of more effective and safer treatment options remains imperative. Therefore, research on natural antioxidants (polyphenols, including ellagitannins such as oenothein B) [4,5] found in naturally grown medicinal plants is relevant globally as they hold the potential to provide alternative solutions to the ongoing challenges of using conventional therapeutic agents with severe side effects during cancer treatment [6,7].

Oxidative stress is a key factor in the development of many diseases, including cancer. The accumulation of reactive oxygen species (ROS) within cells can cause DNA damage, initiate inflammatory responses, and disrupt cellular homeostasis mechanisms recognized as contributors to carcinogenesis. According to Hanahan and Weinberg [8], oxidative stress and ROS-mediated signaling are closely involved in sustaining proliferative signaling, resisting cell death, and promoting genomic instability.

Naturally occurring antioxidants, particularly those derived from plant sources, have therefore attracted considerable interest in their potential to counteract ROS and prevent oxidative damage [9]. Although numerous studies have investigated the advantages of antioxidants obtained from medicinal plants [10,11,12], growing attention has been given to the synergistic interactions among multiple bioactive compounds that may enhance their overall biological activity [13,14]. Further research is necessary to understand the variations in antioxidant activity across different plant species and their respective impact on different cancer cell line types. This relationship suggests that species-specific phytochemical composition and synergistic interactions may influence anticancer responses differently across cell lines, warranting systematic comparative investigation.

Chemical substances with bioactive and antioxidant characteristics, including flavonoids and phenolic acids, are present in different concentrations throughout vegetation stages. To accurately assess their presence in plant extracts, HPLC UV-VIS and HPLC-ED serve as powerful methods for the identification and analysis of antioxidants [15,16]. Integrating chromatographic analyses with in vitro investigations enables a deeper understanding of the interactions between these natural compounds and various types of human and mouse cancer cell lines. Nonetheless, despite an abundance of studies concerning plant-derived antioxidants, there remains no unanimous agreement regarding whether an elevated antioxidant capacity invariably corresponds to enhanced anticancer properties.

Recent studies have reported that extraction methods and solvent composition significantly influence the recovery and bioactivity of phenolic compounds from plant materials [17]. For instance, polar solvents such as methanol, ethanol, and water are commonly used to extract phenolic and flavonoid compounds, and variations in solvent polarity or proportion can significantly affect the extraction efficiency and chemical profile of the resulting extracts [18]. In comparative solvent trials, hydroethanolic mixtures often result in higher phenolic content and stronger antioxidant activity compared to single solvents [19].

Several investigations have also emphasized that the anticancer efficacy of extracts depends not only on total phenolic quantity but also on qualitative composition and interactions among compounds [20]. Synergies within natural product mixtures may enhance biological activity beyond that of isolated compounds [13], a concept reinforced by recent empirical evidence of synergistic antioxidant–anticancer effects in methanolic extracts [21]. However, systematic comparisons across species, extraction methods, and cell types remain limited, which justifies our unified approach.

Our research seeks to fill this gap by investigating the antioxidant properties of multiple plant species to determine their principal bioactive constituents through HPLC UV-VIS and HPLC-ED and evaluating their impacts on five distinct cancer cell lines as well as cytotoxicity on one healthy cell line. Rather than focusing on single compounds, we use plant extracts to reflect the natural complexity of these bioactive mixtures and to determine potential synergistic effects. By analyzing different plant species and biologically active compound concentrations, we aim to gain a deeper understanding of how these variables affect both antioxidant capacity and anticancer activity. Our research is especially significant in the field of natural cancer treatments, as it assists in determining plant-derived compounds that exhibit the greatest potential for subsequent drug or food supplement development. To ensure methodological transparency and enable reproducibility, all procedures were standardized and consistently applied across plant species and assays.

While previous studies have demonstrated a potential link between antioxidant capacity and anticancer efficacy of plant extracts [22], other studies emphasize the importance of molecular mechanisms and compound interactions rather than antioxidant activity alone [23]. Considering these perspectives, this research aims to evaluate the antioxidant potential and anticancer activity of selected medicinal plant extracts and explore possible correlations between antioxidant properties and anticancer activity on various cancer cell lines. This work provides a foundation for future studies on isolating and characterizing the most active biocompounds.

## 2. Materials and Methods

### 2.1. Raw Materials

For this study, 12 medicinal plants (*Urtica dioica* L., *Juglans regia* L., *Juglans nigra* L., *Solidago canadensis* L., *Solidago virgaurea* L., *Quercus robur* L., *Rubus caesius* L., *Fragaria vesca* L., *Symphytum officinale* L., *Ribes nigrum* L., *Rubus idaeus* L., and *Chamaenerion angustifolium* L. Holub) were collected from Kaunas Botanical Garden of Vytautas Magnus University at various stages of vegetation in June–October 2022. The identity of all collected plant samples was confirmed by Dr. Ona Ragažinskienė, a botanist at the Kaunas Botanical Garden of Vytautas Magnus University. Reference specimens were deposited at the Kaunas Botanical Garden Herbarium (KUBG) for future verification. The selection of plant species was based on published data describing their phytochemical composition and biological activities. Species such as *J. nigra* L., *Q. robur* L., and *C. angustifolium* L. have been reported to contain various phenolic compounds, including ellagic acid and its derivatives, associated with antioxidant and anticancer potential [24,25,26,27,28,29,30,31,32,33,34,35,36,37,38,39,40,41,42]. Although *S.* *canadensis* L. does not contain ellagic acid or its derivatives, it was included due to its reported high polyphenolic content and previously described anticancer and antiviral properties [43,44]. Different fresh plant parts were collected and room-dried in the dark for 7–10 days. In total, 21 sample specimens were used in this study.

Table 1 lists all medicinal plants and their parts collected at various stages of vegetation as potential sources of biologically active compounds with activity against cancer cell lines.

The moisture content in each medicinal plant sample was determined by PBM-53 Moisture Balance (Adam Equipment, Kingston, UK) according to the manufacturer’s recommendations.

### 2.2. Chemicals

Methanol (p.a., Chempur, Piekary Śląskie, Poland), sodium carbonate (p.a.; Chempur, Piekary Śląskie, Poland), Folin–Ciocalteu’s phenol reagent (2N; Merck, Darmstadt, Germany), acetic acid (80%; (vol.); Chempur, Piekary Śląskie, Poland), hexamethylenetetramine (99%; Carl Roth, Karlsruhe, Germany), aluminum chloride (99.0%; Alfa Aesar, Karlsruhe, Germany), sodium acetate (99%; Alfa Aesar, Karlsruhe, Germany), DPPH (2,2-diphenyl-1-picrylhydrazyl) (99%; Sigma Aldrich, St. Louis, MO, USA), acetonitrile (99% (vol.); Merck, Darmstadt, Germany), rutin (95%; Merck, Darmstadt, Germany), gallic acid (95%, Thermo Scientific, Waltham, MA, USA), vanillic acid (97%, Sigma Aldrich, St. Louis, MO, USA), ferulic acid (99%, Sigma Aldrich, St. Louis, MO, USA), chlorogenic acid (95%, Sigma Aldrich, St. Louis, MO, USA), ellagic acid (95%, Sigma Aldrich, St. Louis, MO, USA), 2-hydroxycinnamic acid (97%, Sigma Aldrich, St. Louis, MO, USA), 3,4-dihydroxybenzoic acid (97%, Sigma Aldrich, St. Louis, MO, USA), trans-sinapic acid (99%, Sigma Aldrich, St. Louis, MO, USA), trans-p-coumaric acid (99.7%, Sigma Aldrich, St. Louis, MO, USA), dimethyl sulfoxide (99% (vol.); Carl Roth, Karlsruhe, Germany), CellTiter 96^®^ AQ_ueous_ one solution reagent (Promega, Madison, WI, USA), RPMI medium (Thermo Scientific, Waltham, MA, USA), DMEM medium (Thermo Scientific, Waltham, MA, USA), trypsin-EDTA (0.25%, Thermo Scientific, Waltham, MA, USA), L-glutamine (200 mM, Thermo Scientific, Waltham, MA, USA), fetal bovine serum (FBS) (Thermo Scientific, Waltham, MA, USA), penicillin-streptomycin (10,000 U/mL, Thermo Scientific, Waltham, MA, USA), and bidistilled water prepared in the laboratory with a Fistreem Cyclon bidistillator (Cambridge, UK).

### 2.3. Sample Preparation

#### 2.3.1. Extraction Using Organic Solvents

Extracts of 21 sample specimens were prepared from crushed plant parts. The raw material parts were individually crushed using an electric shredder to a particle size of 1–3 mm. Each plant part was weighted (0.5 g) and suspended in 75% (*v*/*v*) methanol in water (20 mL), shaking the mixture in an orbital shaker (Lin-Pro, Labbox labware, Barcelona, Spain) for 24 h at 200 rpm. After extraction, the resulting extracts were filtered through a filter paper and a 0.22 µm membrane filter. The extracts were stored in a refrigerator at 4 °C for further analysis. All extraction procedures were performed under identical controlled conditions (stable room temperature, solvent ratio, and extraction time) to ensure reproducibility and comparability between all tested samples.

#### 2.3.2. Exchange of Extract Solvent for Anticancer In Vitro Testing

There is scientific literature evidence that cancer cells can only tolerate methanol (or ethanol) concentrations of 0.15–2.5% (*v*/*v*) [45]. Since medicinal plant extracts are prepared with a much higher concentration of methanol, it is necessary to remove the solvent to determine the in vitro biological activity of the complex mixture of phenolic and other bioactive compounds present in the extracts against cancer cells. For cell viability assays, and spectrometric and chromatographic analyses, the solvent of the plant extracts was exchanged for dimethyl sulfoxide (DMSO) using a rotary evaporator (Heidolph™ VV Micro, Heidolph Instruments, Schwabach, Germany). An amount of 10 mL of the plant extract was heated in a rotary evaporating flask which was placed in a heated glycerol bath (80 °C) until complete removal of methanol. After heating for 8 min, the remaining part of the extract was dissolved in 2.5 mL 5% (*v*/*v*) DMSO in water. All 21 sample specimens of the plant extracts were stored in a refrigerator at 4 °C for further analysis.

### 2.4. Spectrophotometric Analysis

Total phenolic and flavonoid contents, as well as free radical scavenging activity (RSA) assays, were carried out using a UV-VIS spectrometer from Milton Roy Spectronic (Ivyland, PA, USA). Rutin stock solution (1 mg/mL) was prepared in 75% (*v*/*v*) methanol in water, and working standards were obtained by serial dilution to concentrations of 0.1, 0.3, 0.5, 0.7, and 1.0 mg/mL. The absorbance of rutin was measured at specific wavelengths according to the applied spectrophotometric method, using a reagent blank prepared under the same reaction conditions as for the samples. Each concentration was analyzed in triplicate, and the mean absorbance values were used to construct the calibration curve. A linear relationship was observed between absorbance and concentration, and the resulting regression equation was applied to calculate the total content of phenolic compounds, flavonoids, and radical scavenging activity expressed as mg rutin equivalents (RE) per gram of dry weight. To ensure consistent interpretation across phytochemical and anticancer activity data, IC_50_ values were likewise normalized to rutin equivalents (RE), enabling direct cross-comparison between antioxidant capacity, phenolic composition, and anticancer activity within a unified analytical framework.

The total phenolic content in medicinal plant extracts was determined using the Folin–Ciocalteu method, while flavonoid levels were assessed with the AlCl_3_ colorimetric method—both widely recognized analytical approaches, applied here with slight modifications as described by Bimbiraitė-Survilienė et al. [46]. For phenolic content determination, 100 µL of extract was mixed with 100 µL of 2N Folin–Ciocalteu reagent and 3000 µL of 4% (*v*/*v*) sodium carbonate solution. After 30 min of incubation at room temperature, the absorbance was measured at 760 nm. Flavonoid content was determined by mixing 80 µL of extract with an AlCl_3_ reagent prepared in methanol containing acetic acid, hexamethylenetetramine, and aluminum chloride, followed by 30 min of incubation at 4 °C. The absorbance was measured at 407 nm. Antioxidant activity was evaluated using a DPPH radical scavenging assay [15], in which 77 µL of extract was added to 3000 µL of DPPH solution (100 mM). After 15 min of incubation in the dark at room temperature, the absorbance was measured at 515 nm. All measurements were performed in triplicate.

### 2.5. HPLC UV-VIS System Analysis

The separation and identification of phenolic compounds were performed using a modular HPLC system, composed of a binary mobile phase pump (Agilent 1100 HPLC G1312A, Santa Clara, CA, USA), a manual injector (Rheodyne™ 7725, Santa Clara, CA, USA), a chromatography column (125 mm × 4 mm, LiChrospher^®^ 100 RP-18, particle size 5 µm (Merck, Darmstadt, Germany), and a UV detector (Linear UVIS 200, Waltham, MA, USA). Data was processed using a dedicated DataApex Clarity Lite software system (version 2.6.6.574). Samples were injected using a 25 µL Hamilton^®^ syringe (model 1702 RNR). The mobile phases consisted of bidistilled water acidified with 0.05% (*v*/*v*) trifluoracetic acid (A) and pure methanol acidified with 0.05% (*v*/*v*) trifluoracetic acid (B). The following gradient elution program was applied: from 0 to 10 min, the concentration of component B increased from 0% to 25%, from 10 to 38 min, from 25% to 60%, from 38 to 40 min, and from 60% to 95%, which was held for an additional 5 min. The flow rate was maintained at 0.75 mL/min, and the injection volume was 20 µL. A 265 nm wavelength was used. Compounds were determined based on their retention times using the external standard method. Retention times of standard compounds were as follows: gallic acid—5.03 min, 3,4-dihydroxybenzoic acid—6.94 min, vanillic acid—10.66 min, chlorogenic acid—10.75 min, caffeic acid—11.74 min, syringic acid—12.59 min, trans-p-coumaric acid—17.15 min, ferulic acid—18.67 min, trans-sinapic acid—19.84 min, salicylic acid—21.08 min, 2-hydroxycinammic acid—22.44 min, rutin—27.18 min, trans-cinnamic acid—28.47 min, and ellagic acid—28.63 min.

### 2.6. HPLC-ED System Analysis

To separate and detect antioxidants in medicinal plant extracts, we used a gradient HPLC system (ESA, Chelmsford, MA, USA), operated with two ESA 582 HPLC pumps to generate a high-pressure gradient.

Twelve external standards—3,4-dihydrobenzoic acid, trans-p-coumaric acid, chlorogenic acid, gallic acid, caffeic acid, syringic acid, rutin, vanillic acid, trans-sinapic acid, ferulic acid, ellagic acid, and 2-hydroxycinammic acid—were selected as external standards. Each standard was individually injected into the HPLC-ED system to determine the retention times and, using the external standard method, determine the compounds in plant extracts. Antioxidants were registered using an electrochemical detector cell array operated at 300, 500, and 700 mV potentials. Among all potentials, the highest peak in only one cell was chosen. The concentration of each determined compound was expressed in rutin equivalents (RE) in mg of rutin per gram of dry material, calculated using a rutin calibration curve.

The HPLC-ED analysis was carried out following our previous studies [15,16]. A 10 µL sample was manually injected using a 25 µL Hamilton^®^ syringe (model 1702RNR). Separation was carried out on a reversed-phase C18 chromatography column (125 mm × 4 mm), LiChrospher^®^ 100 RP-18e, particle size 5 µm (Merck, Darmstadt, Germany). Detection was performed using a model 5600 CoulArray electrochemical detector equipped with an array of cells set at 300, 500, and 700 mV. The mobile phase flow rate was consistently maintained at 0.4 mL/min. Elution was achieved using a two-component mobile phase. Solvent A consisted of 50 mM sodium dihydrogen phosphate (pH 3) with 1% (*v*/*v*) methanol, while Solvent B was a mixture of 100 mM sodium dihydrogen phosphate (pH 3), acetonitrile, and methanol (30:60:10 (*v*/*v*)). The gradient profile started from 25% of Solvent B at 5 min, increased to 60% by 38 min, increased to 95% at 45 min, and returned to 5% at 58 min and remained at 5% until the end of the equilibration of the column at 60 min. Throughout the analysis, the flow rate was set at 0.4 mL/min. Data was processed using a dedicated CoulArray^®^ software system (version 1.02).

### 2.7. Medicinal Plant Extract Effect on Cell Viability

#### 2.7.1. Cell Lines

Five cancer cell lines were used for medicinal plant extract specimens to determine their effect on cell viability and bioactivity assessment: 4T1 (a breast cancer cell line derived from mammary gland tissue of a mouse), A549 (adenocarcinoma human alveolar basal epithelial cell line), Caki-1 (a human kidney carcinoma cell line), HCT116 (a human colon cancer cell line), and MCF7 (a human breast cancer cell line). A selection of different cancer cells was made to test the same extracts against various cancers. In this study, the cytotoxicity of plant extracts was also determined using a healthy human embryonic kidney cell line—HEK-293. This cell line was selected as representative of a non-cancerous control because it is widely used in cytotoxicity studies as a model of healthy human cells due to its well-characterized nature, reproducibility, and ease of culture [47]. All cell lines used in this study are immortalized cell lines obtained from the American Type Culture Collection (ATCC, Manassas, VA, USA). Cells were revived from frozen stocks and routinely cultured under standard conditions prior to experimentation. The cell lines were grown as adherent 2D monolayers in Petri dishes in RPMI containing 10% FBS, 1% penicillin-streptomycin or DMEM containing 10% FBS, 1% penicillin-streptomycin, and 1% L-glutamine nutrient media, depending on the cell type. Cancer cell lines were cultured for 2 to 3 weeks after thawing them from liquid nitrogen with the following protocol: 0.3–0.4 million cell lines were cultured every 2 to 3 days in 10 mL nutrient media until stable morphological cell growth was achieved (1.8–2.2× cell duplication per 24 h).

#### 2.7.2. MTS Cell Proliferation Assay

The MTS assay is a well-established colorimetric method used to evaluate cell metabolic activity as an indicator of cell viability [48,49]. The MTS assay protocol is based on the reduction in the MTS (3-(4,5-dimethylthiazol-2-yl)-5-(3-carboxymethoxyphenyl)-2-(4-sulfophenyl)-2H-tetrazolium) tetrazolium compound (bright yellow) by viable cells to generate a colored formazan dye (dark brown) that is soluble in cell culture media.

Cell cultures were cultivated in 96-well plates. To ensure consistent cell numbers after exposure to different plant extracts, cell number is cultivated in each well: 8000 cells per well for the 24 h test, 4000 cells per well for the 48 h test and 2000 cells per well for the 72 h test. This approach ensured comparable cell densities at the time of extract treatment and measurement, since cell proliferation approximately doubled every 24 h. Each well was supplemented with 1–18 µL of plant extract and 182–199 µL of RPMI or DMEM culture medium containing cancer cell lines, bringing the total volume to 200 µL per well. The positive control consisted of wells with cancer cell lines and culture medium without plant extracts (total volume: 200 µL), allowing for baseline comparison of cell viability. The negative control (background) contained only culture medium (total volume: 200 µL), ensuring the accuracy of absorbance measurements. Additionally, a 5% DMSO (*v*/*v*) control was used to assess if DMSO, as a solvent, had any negative effects on cell growth. While no standard chemotherapeutic agent was included as a reference compound, the primary objective was to evaluate the inherent anticancer activity potential of the tested plant extracts rather than benchmark them against existing anticancer drugs. The 96-well plates were incubated at 37 °C with 6.0% CO_2_ concentration for 24, 48, and 72 h. All MTS assays were conducted using identical culture conditions, incubation parameters, and reagent concentrations to ensure reproducibility and comparability of results across all tested extracts and cell lines.

After incubation, the whole culture medium with cell lines and plant extracts was removed and replaced with fresh 100 µL RPMI or DMEM medium and 20 µL CellTiter 96^®^ AQ_ueous_ with one solution reagent per well. The plates were then incubated for 3 h at 37 °C with a 6.0% CO_2_ concentration. After an incubation period, absorbance at 492 nm was measured using the Biosan HiPo MPP-96 microplate photometer (Riga, Latvia). A dose–response curve was plotted with extract concentrations (RE mg/g) on the x-axis and cell viability (%) on the y-axis. A regression equation was used to determine the extract concentration required to inhibit 50% of cell growth. IC_50_ values are expressed as rutin equivalents (RE) in mg of rutin per gram of dry material following spectrometric analysis results or radical scavenging activity to have uniformity across study results.

### 2.8. Statistical Analysis

The experimental data were analyzed using SPSS Statistics for Windows, version 30.0.0 (SPSS Inc., Chicago, IL, USA). All assays were conducted in triplicate (n = 3), with results expressed as mean values ± standard deviation (SD).

For cell viability (IC_50_) measurements, data were obtained from DMSO-based extracts at 24 h, 48 h, and 72 h incubation across five cancer cell lines (4T1, A549, Caki-1, HCT116 and MCF7), where lower values indicate stronger anticancer activity. Because IC_50_ values were only obtained from DMSO medicinal plant extracts, no direct statistical comparison between solvents was performed. Instead, Pearson correlation analyses were carried out at each incubation period between IC_50_ values and extract composition parameters. All tests were two-tailed, and *p*-values < 0.05 were considered statistically significant. For correlation analyses between IC_50_ values and extract composition parameters, a Bonferroni correction was applied to account for multiple comparisons (α = 0.002 for 25 tests per timepoint). IC_50_ values were evaluated separately for each incubation period (24 h, 48 h, and 72 h). Differences across timepoints were described qualitatively based on observed trends, without formal statistical comparison.

## 3. Results

### 3.1. Determination of Total Phenolic Content, Flavonoid Content, and Antioxidant Activity by Spectrometric Methods

**Figure 1:**
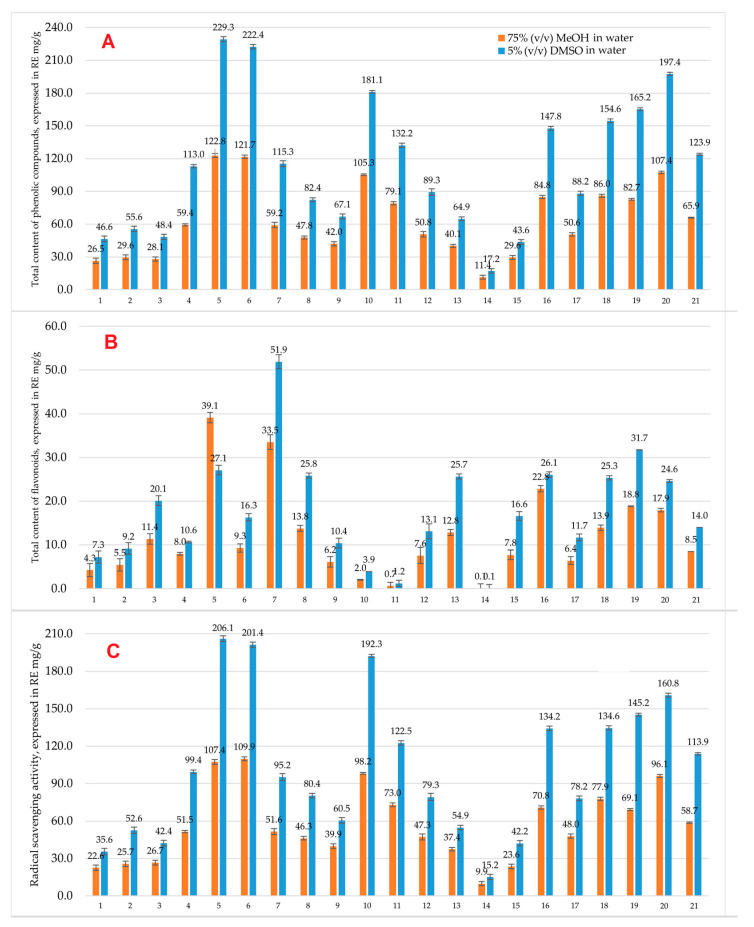
Total content of phenolic compounds (**A**), total content of flavonoids (**B**), and radical scavenging activity (**C**), determined in medicinal plant specimens using methanolic and DMSO-based extracts, expressed in RE mg/g (n = 3). Legend: 1—Stinging nettle (aerial part); 2—Common walnut (pericarp); 3—Common walnut (leaves); 4—Common walnut (bark); 5—Black walnut (pericarp); 6—Black walnut (fruit); 7—Canadian goldenrod (aerial part); 8—European goldenrod (aerial part); 9—Pedunculate oak (leaves); 10—Pedunculate oak (fruit); 11—Pedunculate oak (bark); 12—European dewberry (leaves); 13—Wild strawberry (leaves); 14—Comon comfrey (roots); 15—Blackcurrant (leaves); 16—Raspberry (leaves); 17, 18, 19, 20, 21—Fireweed (aerial part).

Figure 1 illustrates the total phenolic compound content (A), total content of flavonoids (B), and radical scavenging activity (C) in all medicinal plant specimens. The moisture content of room-dried samples ranged from 3.44% to 8.37%, confirming adequate and consistent drying prior to extraction. The water mass fraction was considered in all calculations to express results on a dry weight basis. All samples were extracted with 75% (*v*/*v*) methanol in water, hereafter referred to as methanolic extract. To enable cell-based testing, methanol was replaced by solvent exchange, and the residues were re-dissolved in 5% (*v*/*v*) DMSO in water, referred to as the DMSO-based extract. Results are expressed in rutin equivalents (RE) mg of rutin per gram of dry material (mg/g).

As can be seen in Figure 1, the highest contents of phenolic compounds were determined in black walnut (*J. nigra* L.) pericarp and fruit samples during the ripening vegetation stage, resulting in 122.8 ± 3.12 and 121.7 ± 2.95 RE mg/g, respectively. These results indicate that the total content of phenolic compounds was comparable between different plant parts. The second largest amount of phenolic content was determined in fireweed (*C. angustifolium* L.), specifically during the massive flowering vegetation stage, resulting in 107.4 ± 2.45 RE mg/g. The third highest value of total content of phenolic compounds was determined in pedunculate oak (*Q. robur* L.) fruits during the ripening vegetation stage, resulting in 105.3 ± 3.21 RE mg/g. After exchanging the solvents, all medicinal plant extracts became more concentrated. For methodological reasons, methanol was evaporated and the remaining residue was dissolved in 5% (*v*/*v*) DMSO in water, resulting in a final volume that was half the original. As a result, the extracts were approximately twice as concentrated. DMSO as a solvent was chosen due to its good solubility for phenolic compounds as well as being compatible with cell viability assays. As demonstrated in Figure 1, black walnut (pericarp and fruit), fireweed (aerial part), and pedunculate oak (fruit) medicinal plant extracts had higher total content of phenolic compounds that increased by 86.7%, 82.7%, 83.8%, and 71.9%, respectively.

There were several medicinal plant extracts that had a high total content of flavonoids when compared to the rest of the plant extracts. The highest number of flavonoids was observed in black walnut (*J. nigra* L.) pericarp, resulting in 39.1 ± 1.2 RE mg/g. The second-highest total content of flavonoids was observed in the Canadian goldenrod (*S. canadensis* L.) sample, resulting in 33.5 ± 0.5 RE mg/g. Another medicinal plant sample that resulted in high flavonoid content was raspberry (*R. idaeus* L.), resulting in 22.8 ± 0.2 RE mg/g. After solvent exchange, total contents of flavonoids were similar in both methanolic and DMSO-based extracts. This was expected because flavonoids are less polar than phenolic acids and, as a result, they are less soluble in 5% (*v/v*) DMSO in water. However, in some cases, solvent exchange did increase flavonoid content in medicinal plants. In Canadian goldenrod and raspberry samples, total content of flavonoids increased by 54.9% and 14.5%, respectively. In other cases, like black walnut’s pericarp specimen, total content of flavonoids decreased by 30.7%, indicating poor solubility or poor high-temperature stability. In the fireweed sample, total content of flavonoids in DMSO-based extract increased by 68.6%, although it showed low initial content in the methanolic extract.

The highest radical scavenging activity (RSA) was determined in black walnut, both pericarp and fruit, as well as pedunculate oak (fruit) and fireweed (aerial part, massive flowering vegetation stage) samples. Radical scavenging activity resulted in 107.4 ± 4.7 RE mg/g, 109.9 ± 5.1 RE mg/g, 98.2 ± 2.5 RE mg/g, and 96.1 ± 4.1 RE mg/g, respectively. In these medicinal plant samples, radical scavenging activity increased by 91.9%, 83.3%, 92.5%, and 67.3%, indicating that samples were concentrated after solvent exchange. Pearson correlation analysis demonstrated a very strong correlation (r = 0.993, *p* < 0.001) between RSA and total content of phenolic compounds across all medicinal plant extracts. This indicates that the antioxidant capacity of the studied extracts is primarily driven by their phenolic compound content. In practical terms, this relationship suggests that optimizing extraction methods to maximize phenolic compound yield can directly enhance the RSA and potential bioactive properties of the extracts.

### 3.2. HPLC Analysis

#### 3.2.1. 75% (*v*/*v*) Methanol in Water Extracts

To perform a comparative quantitative analysis of phenolic compounds found in 21 medicinal plant samples and extracted using 75% (*v*/*v*) methanol in water, a HPLC UV-VIS and HPLC-ED was used. The ultraviolet–visible detector enabled quantification of a broad range of compounds containing UV-absorbing chromophores [50], while electrochemical detection quantified electroactive compounds with redox-active functional groups [51]. This dual-detection approach was used to assess the concentration levels and detection selectivity of key bioactive compounds. All quantitative results obtained from both HPLC UV-VIS and HPLC-ED analyses were expressed as mg/g of dry material to ensure comparability across samples and detection methods. The results obtained by both methods are shown in Table 2. Because not all phenolic compounds exhibit both UV absorbance and electrochemical activity, the use of both HPLC UV-VIS and HPLC-ED was necessary to achieve comprehensive detection coverage. UV-VIS enabled identification of non-redox-active compounds, while ED enhanced sensitivity for redox-active phenolics.

Among the 21 medicinal plant samples analyzed, ellagic acid was detected in several species—*Fagaceae* (*Q. robur* L.), *Juglandaceae* (*J. regia* L.), *Rosaceae* (*R. caesius* L., *F. vesca* L.), and *Onagraceae* (*C. angustifolium*)*,* primarily in leaf, fruit, and bark samples. However, ellagic acid was not detected in all plant samples, suggesting species-specific differences in ellagic acid content, its chemical form (either free or bound in ellagitannins), or concentrations below the detection limit. Although in several cases ellagic acid peak areas appeared higher in HPLC-ED, the quantification obtained by HPLC UV-VIS was considered more reliable, as the UV signal showed better reproducibility and linearity across the calibration range. In contrast, HPLC-ED was less consistent, likely due to ellagic acid’s high oxidation potential and limited electrochemical reactivity under applied potentials, which caused variable or weak detector responses. Consequently, ellagic acid contributed strongly to total UV-VIS peak area but not consistently in HPLC-ED chromatograms, reflecting its limited redox behavior under standard analytical conditions.

A related compound, oenothein B, a macrocyclic ellagitannin and derivative of ellagic acid precursors, was identified in high concentrations in fireweed (*C. angustifoium* L.). This compound showed very strong UV absorbance, resulting in one of the highest individual peak areas in HPLC UV-VIS, and was also detectable by HPLC-ED, likely due to the partial oxidation of its multiple polyphenolic subunits or degradation into smaller electroactive fragments. The dual detectability of oenothein B contributed substantially to the overall chromatographic signal in both HPLC methods. This highlights oenothein B’s analytical and biological relevance, as well as the advantage of combining both detection methods when targeting complex polyphenolic compounds.

In addition to ellagic acid and oenothein B, several other phenolic compounds were frequently detected across the medicinal plant extracts. Gallic acid, caffeic acid, and 3,4-dihydroxybenzoic acid were consistently identified in multiple samples and showed strong signals in both UV-VIS and ED due to their well-known chromophores and electroactive functional groups. In contrast, a compound like trans-cinnamic acid was primarily detected only using the ultraviolet–visible detector, reflecting the compound’s poor electrochemical reactivity. Although only 12 compounds were identified and quantified, many additional peaks were detected in both HPLC chromatograms, particularly in UV-VIS, indicating the presence of other phenolic compounds that could not be identified due to the absence of reference standards. These unidentified compounds contributed significantly to the total peak area, especially in UV-VIS, which explains why this method consistently yielded higher cumulative signal intensities than HPLC-ED.

To assess the consistency between both detection methods, Pearson correlation coefficients were calculated between total peak areas, total content of phenolic compounds, and RSA. A very strong positive correlation was found between HPLC UV-VIS and HPLC-ED total peak areas (r = 0.905, *p* < 0.001), confirming that both methods capture similar compound patterns across plant species. Both detection methods also showed moderate but statistically significant correlations with total content of phenolic compounds, total content of flavonoids, and radical scavenging activity. These results confirm that despite their differences in detection, both methods provide reliable and complementary quantitative data that can be used in combination for phytochemical profiling and bioactivity screening of medicinal plant extracts.

#### 3.2.2. Extracts After Solvent Exchange to 5% (*v*/*v*) DMSO in Water

To evaluate the recovery and solubility of phenolic compounds in an alternative solvent system, the methanolic extracts were subjected to solvent exchange by evaporating methanol and re-dissolving the remaining residue in 5% (*v*/*v*) DMSO in water. The same HPLC UV-VIS and HPLC-ED analytical methods were applied to assess whether the target compound remained present and detectable after solvent exchange. The quantitative data obtained from the 5% (*v*/*v*) DMSO in water extracts are presented in Table 3.

The results of the DMSO-based extracts prepared in 5% (*v*/*v*) DMSO in water confirmed that the majority of previously identified phenolic compounds were successfully recovered, and in several cases, showed increased concentrations compared to the original methanolic plant extracts. Similarly, compounds such as ellagic acid, oenothein B, gallic acid, and 3,4-dihydroxybenzoic acid were consistently detected with high concentrations in both HPLC UV-VIS and HPLC-ED analyses, particularly in *C. angustifolium* L. and *Q. robur* L. While the qualitative compound profile remained largely unchanged, total peak areas were generally higher in the DMSO-based extracts, especially in HPLC-ED chromatograms, supplementing the spectrometric analysis results that indicate extract concentration after solvent exchange.

### 3.3. Cell Viability to Medicinal Plant Extracts

Table 4 shows the half-maximal inhibitory concentration (IC_50_) values of plant extracts against different cancer cell lines tested, expressed in RE mg/g.

As can be observed in Table 4, different medicinal plant samples inhibited the viability of five cancer cell lines differently. The lowest IC_50_ value that was obtained to inhibit mouse breast cancer cell line 4T1 viability by 50% after 24 h of incubation time was determined using Canadian goldenrod (*S. canadensis* L.) extract, resulting in the IC_50_ value being 2.92 ± 0.05 RE mg/g. The second and third medicinal plants demonstrated slightly higher IC_50_ values and were determined using blackcurrant (*R. nigrum* L.) and fireweed (*C. angustifolium* L.) extracts, resulting in IC_50_ values being 3.48 ± 0.11 RE mg/g and 3.80 ± 0.07 RE mg/g, respectively. After 48 h of incubation, the same plants—fireweed, blackcurrant and Canadian goldenrod—demonstrated the lowest IC_50_ values, resulting in 1.28 ± 0.06 RE mg/g, 1.36 ± 0.05 RE mg/g, and 1.38 ± 0.07 RE mg/g, respectively. After 72 h of incubation, the 4T1 cancer cell line with different medicinal plant extracts showed slightly different results. Two fireweed samples, collected during intensive growth and flower bud development vegetation stages, and pedunculate oak (*Q. robur* L.) leaf samples demonstrated the lowest IC_50_ values, being 0.92 ± 0.01 RE mg/g, 0.28 ± 0.01 RE mg/g, and less than 0.84 ± 0.02 RE mg/g, respectively.

Different results were determined using adenocarcinoma human alveolar basal epithelial cell line A549. After 24 h incubation with different plant extracts, pedunculate oak (*Q. robur* L.) leaf and fruit and common walnut (*J. regia* L.) bark samples demonstrated the lowest IC_50_ values, resulting in 1.04 ± 0.03 RE mg/g, 1.24 ± 0.01 RE mg/g, and 1.36 ± 0.02 RE mg/g, respectively. The same samples demonstrated the lowest IC_50_ values after 48 h incubation, resulting in 1.16 ± 0.02 RE mg/g, 0.92 ± 0.07 RE mg/g, and 1.20 ± 0.02 RE mg/g, respectively. After 72 h incubation, stinging nettle (*U. dioica* L.), the same common walnut bark and fireweed (*C. angustifolium* L.) samples demonstrated the lowest IC_50_ values, being less than 0.36 ± 0.01 RE mg/g, 0.40 ± 0.01 RE mg/g, and 0.40 ± 0.02 RE mg/g, respectively.

Medicinal plant extract samples inhibited human kidney carcinoma cell line Caki-1 differently when compared to 4T1 and A549 cancer cell lines. After 24 h incubation, common walnut (*J. regia* L.) pericarp, Canadian goldenrod (*S. canadensis* L.), and black walnut (*J. nigra* L.) fruits demonstrated the lowest IC_50_ values, resulting in 1.60 ± 0.02 RE mg/g, 3.16 ± 0.01 RE mg/g, and 3.16 ± 0.05 RE mg/g, respectively. After 48 h incubation, the same common walnut and black walnut samples, as well as pedunculate oak (*Q. robur* L.) fruits, indicated the lowest IC_50_ values, resulting in 2.00 ± 0.05 RE mg/g, less than 1.56 ± 0.01 RE mg/g, and less than 1.76 ± 0.07 RE mg/g, respectively. Similarly to 48 h incubation, after 72 h, the same common and black walnut samples, as well as pedunculate oak, demonstrated the lowest IC_50_ values, being 1.48 ± 0.05 RE mg/g, less than 1.56 ± 0.02 RE mg/g, and less than 1.64 ± 0.06 RE mg/g, respectively.

Unlike the previous three cancer cell lines, the human colon cancer cell line HCT116 was more aggressive and demonstrated greater survival. In some cases, medicinal plant extracts were unable to demonstrate any anticancer activity against the HCT116 cell line. However, some plant samples did inhibit cancer cell line growth, and after 24 h incubation, black walnut (*J. nigra* L.) pericarp, Canadian goldenrod (*S. canadensis* L.), and fireweed (*C. angustifolium* L.) aerial part samples, collected during massive flowering vegetation stage, demonstrated the lowest IC_50_ values, being 5.28 ± 0.05 RE mg/g, 6.96 ± 0.010 RE mg/g, and 9.88 ± 0.05 RE mg/g, respectively. The same samples after 48 h incubation demonstrated the lowest IC_50_ values, being less than 2.56 ± 0.02 RE mg/g, 4.20 ± 0.01 RE mg/g, and 3.92 ± 0.09 RE mg/g, respectively. After 72 h incubation, black walnut pericarp and fruit, as well as fireweed aerial part, collected during the flower bud development vegetation stage, demonstrated the lowest IC_50_ values, being 2.56 ± 0.06 RE mg/g, 2.52 ± 0.05 RE mg/g, and 2.96 ± 0.08 RE mg/g, respectively.

The most aggressive line, demonstrating the best survival against plant extracts, was the human breast cancer cell line MCF7. A total of 4 samples out of 21 could not demonstrate any inhibition against this cancer cell line. However, the rest of the sample demonstrated some cell growth inhibition. Most of the IC_50_ values after 24 h incubation were between 10.0 and 20.0 RE mg/g. However, Canadian goldenrod (*S. canadensis* L.), common walnut (*J. regia* L.) pericarp, and fireweed (*C. angustifolium* L.) aerial part samples, collected during the massive flowering vegetation stage, demonstrated the lowest IC_50_ values, being 4.04 ± 0.01 RE mg/g, 7.76 ± 0.02 RE mg/g, and 8.24 ± 0.15 mg/g, respectively. After 48-hour incubation, the same Canadian goldenrod and fireweed samples, as well as common walnut leaves, indicated the lowest IC_50_ values, resulting in 3.08 ± 0.06 RE mg/g, 6.60 ± 0.16 RE mg/g, and 4.72 ± 0.01 RE mg/g. Similarly to 48-h incubation, after 72 h, Canadian goldenrod and fireweed, as well as black walnut pericarp, demonstrated the lowest IC_50_ values, being 2.16 ± 0.01 RE mg/g, 2.32 ± 0.06 RE mg/g, and 3.96 ± 0.06 RE mg/g, respectively.

The difference in IC_50_ values among extracts can be partly explained by variations in their phenolic profiles. Medicinal plant extracts rich in ellagic acid and oenothein B (i.e., *C. angustifolium* and *Q. robur* L.) demonstrated the strongest anticancer activity with their high total phenolic content. Similarly, elevated levels of gallic acid and caffeic acid in *J. regia* L. and *S. canadensis* L. extracts were associated with low IC_50_ values, supporting the contribution of these compounds to anticancer activity.

To provide a comprehensive overview of statistical analyses, all key correlation results are summarized in Table 5. The table compiles Pearson correlation coefficients (r), *p*-values, and incubation times, highlighting the relationship between phytochemical content, antioxidant capacity, HPLC detection methods, and anticancer activity across different incubation times and cancer cell lines.

Analysis of the phytochemical composition revealed clear patterns of inter-correlation. Antioxidant activity, measured by radical scavenging activity, was found to correlate very strongly with total content of phenolic compounds, highlighting the dominant role of phenolic compounds in the antioxidant capacity of medicinal plant extracts. Both HPLC UV-VIS and HPLC-ED methods demonstrated significantly moderate correlations with total content of phenolic compounds, total content of flavonoids, and radical scavenging activity. When comparing both HPLC detection methods, a very strong significant correlation was observed, confirming these methods’ reliability and complementarity in profiling bioactive compounds.

The correlation analysis revealed several important trends in the relationship between phytochemical composition and anticancer activity. Total content of phenolic compounds, radical scavenging activity, and HPLC UV-VIS and HPLC-ED total peak area showed multiple nominal correlations with IC_50_ values, particularly at 48 h incubation, but none of these survived Bonferroni correction. In contrast, two inter-cell correlations remained statistically significant after Bonferroni adjustment: IC_50_ (4T1) correlated strongly with IC_50_ (HCT116) at 24 h incubation (r = 0.673, *p* < 0.001) and with IC_50_ (A549) at 48 h incubation (r = 0.661, *p* = 0.001). These results indicate consistent patterns of sensitivity among these (4T1, HCT116, and A549) cancer cell lines. Additional uncorrected correlations (*p* < 0.05) were observed, including negative correlations between 4T1 IC_50_ values and total content of phenolic compounds, radical scavenging activity, and HPLC measures at 48 h incubation, as well as positive correlations between MCF7 IC_50_ values and multiple phytochemical variables at 24 h and 48 h incubations. Negative correlations indicate that higher phytochemical or antioxidant levels are linked to stronger anticancer activity (lower IC_50_), whereas positive correlations suggest that higher phytochemical content corresponded to weaker activity (higher IC_50_). While several nominal correlations were observed, those not surviving Bonferroni correction should be interpreted cautiously, as they may represent false-positive associations due to multiple tests.

Time-dependent differences were also observed. At 24 h incubation, only a few nominal correlations were detected. By 48 h incubation, stronger and more consistent correlations emerged, particularly for the 4T1 cancer cell line, where higher phenolic content and antioxidant activity tended to correlate with increased anticancer activity (lower IC_50_). In contrast, the MCF7 cancer cell line often showed inverse or positive correlations at later incubation, suggesting weaker or opposite relationships with phytochemical content. At 72 h incubation, fewer correlations remained, though some inter-cell correlations and inverse relationships between Caki-1 and MCF7 remained, indicating divergent cell line-specific responses with prolonged exposure.

To highlight the extracts with the highest anticancer activity, we calculated the estimated anticancer potential per gram of dried medicinal plant material based on IC_50_ values of each cell line and incubation time. Since IC_50_ values are specific to the extracts, this normalization reflects the relative bioactive potential of the raw plant material expressed on a dry weight basis, rather than a direct count of cell death. Table 6 below shows extracts with the highest anticancer activity potential, expressed as a range (minimum–maximum) of cancer cells killed (in millions), indicating their potency.

Table 6 represents the highest anticancer activity extracts based on the number of cancer cells killed per gram (×10^6^) of dry plant material across five cancer cell lines during 24, 48, and 72 h incubation. The results clearly determine time-dependent increases in anticancer activity effectiveness for several plant species. Fireweed (*C. angustifolium* L.) exhibited the highest activity, killing between 104.14 and 190.14 million 4T1 cells per gram after 72 h and showing significant anticancer activity against the Caki-1 cancer cell line (11.57–21.13 million). Stinging nettle (*U. dioica* L.) followed closely, killing up to 147.89 million 4T1 cells after 72 h incubation. At an earlier incubation time, Canadian goldenrod (*S. canadensis* L.) was effective against 4T1 and MCF7 cells (up to 15.07 and 10.89 million, respectively), while pedunculate oak (*Q. robur* L.) leaves and fruits showed strong anticancer activity against A549 cells (up to 52.61 million) at 24- and 48-hour incubation. Black walnut (*J. nigra* L.), particularly its pericarp, showed consistent anticancer activity toward HCT116 cells, reaching up to 18.91 million cells killed.

Additionally, to facilitate the presentation of our results, we include a representative dose–response curve below for one of the medicinal plant extract effects of all cell lines tested, demonstrating the typical response pattern. Due to the large number of data points, it is not feasible to display all curves. However, the complete IC_50_ values are provided in Table 4 for a comprehensive comparison.

**Figure 2:**
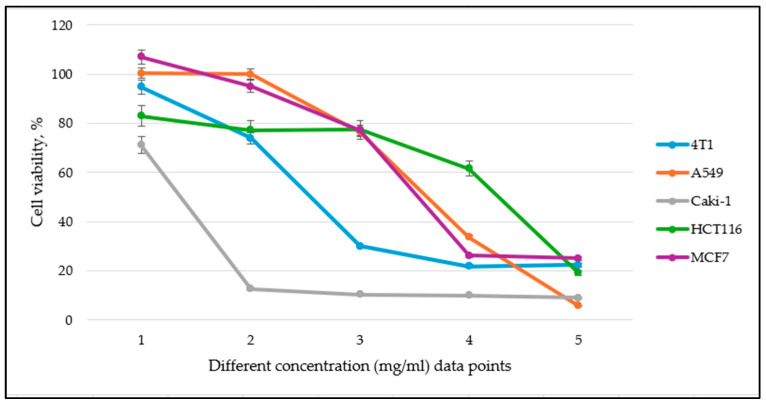
Cell viability (%) after 24 h incubation across different concentrations for 4T1, A549, Caki-1, HCT116, and MCF7 cancer cell lines used to determine IC_50_ values of the Pedunculate oak (*Q. robur* L.) extract.

Figure 2 represents the effect of varying concentrations of the tested Pedunculate oak (*Q. robur* L.) extract on the viability of 4T1, A549, Caki-1, HCT116, and MCF7 cell lines. The x-axis represents different concentration data points (mg/ml), which vary among cell lines due to the need for adjustments to determine IC_50_ values accurately. Specifically, for the A549 cell line, lower concentrations were used to observe inhibitory effects, while for MCF7, higher concentrations were required to achieve a measurable decrease in viability. The y-axis represents the percentage of viable cells relative to the control.

In this study, we also tested cytotoxic effects against the human embryonic kidney healthy cell line HEK-293, which is treated as a healthy model cell line, specifically demonstrating different compound cytotoxicity to healthy mammalian cells. The same MTS assay protocol was used with the HEK-293 healthy cell line, which resulted in all cell viability being 90.0–100.0%, indicating no cytotoxic effect, preventing the determination of IC_50_ values.

**Figure 3:**
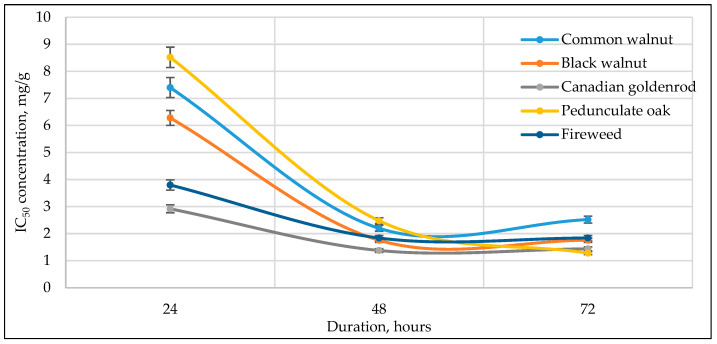
Time-dependent IC_50_ values of different plant extracts against 4T1 cancer cell line at 24, 48, and 72 h, indicating anticancer activity kinetics.

**Figure 4:**
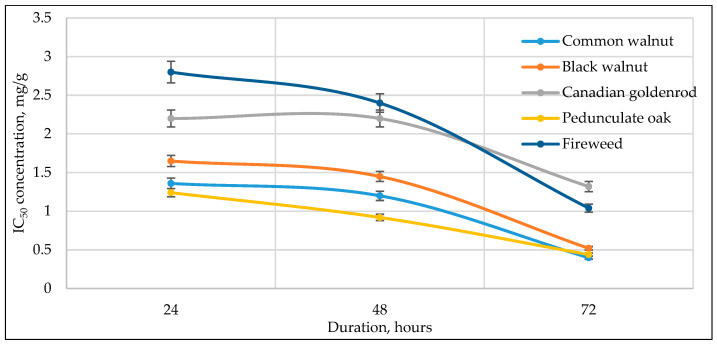
Time-dependent IC_50_ values of different plant extracts against A549 cancer cell line at 24, 48, and 72 h, indicating anticancer activity kinetics.

**Figure 5:**
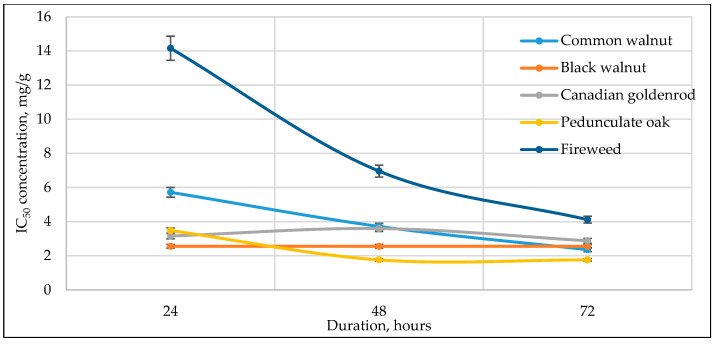
Time-dependent IC_50_ values of different plant extracts against Caki-1 at 24, 48, and 72 h, indicating anticancer activity kinetics.

**Figure 6:**
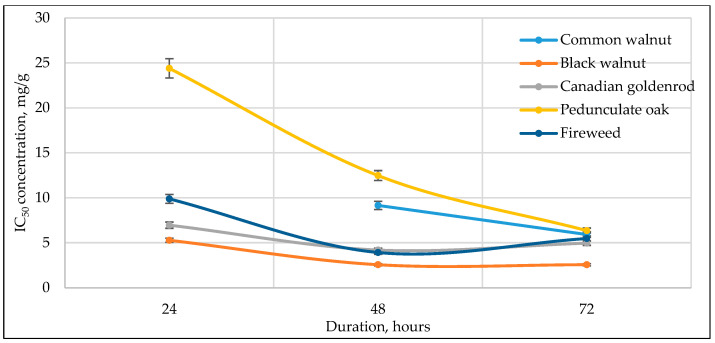
Time-dependent IC_50_ values of different plant extracts against HCT116 cancer cell line at 24, 48, and 72 h, indicating anticancer activity kinetics.

**Figure 7:**
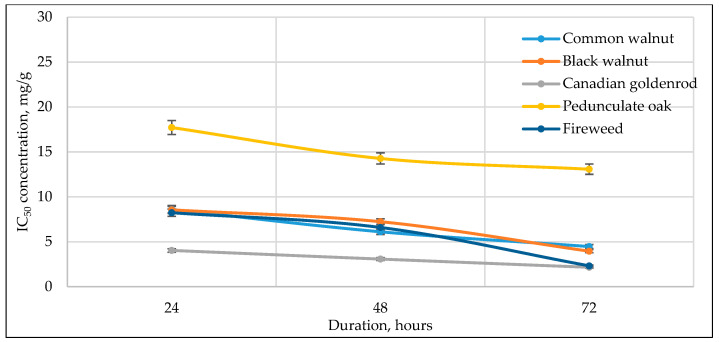
Time-dependent IC_50_ values of different plant extracts against MCF7 cancer cell line at 24, 48, and 72 h, indicating anticancer activity kinetics.

Figure 3, Figure 4, Figure 5, Figure 6 and Figure 7 below show the time-dependent anticancer activity kinetics of varying highest anticancer activity shown in plant extracts on various cancer cell lines based on IC_50_ values at 24, 48, and 72 h.

As can be seen in Figure 3, Figure 4, Figure 5, Figure 6 and Figure 7, across all cancer cell lines, IC_50_ values generally decrease over time, indicating increasing anticancer activity with longer exposure. Canadian goldenrod and fireweed consistently demonstrated the highest anticancer activity (lowest IC_50_ values), particularly against 4T1, HCT116, and A549 cells. Pedunculate oak starts with high IC_50_ (low anticancer activity) but decreases significantly over time, suggesting delayed yet strong effects or longer incubation periods for anticancer activity, especially in HCT116 and Caki-1 cancer cell lines. Common walnut demonstrates moderate anticancer activity but shows an increase in IC_50_ in some cases, indicating reduced long-term effectiveness. Black walnut samples generally have strong anticancer activity, though some fluctuations suggest varying potency across cell lines.

## 4. Discussion

The study aimed to determine how different medicinal plants and their parts, i.e., aerial part, pericarp, fruit, leaves, and bark, contain biologically active compounds that can indicate different human and mouse cancer cell line viability and possible cytotoxicity to healthy human cell lines. Comparing the total content of phenolic compounds, RSA, determined antioxidants, and different cell line growth inhibitions results, this study highlights preliminary evidence of the use of these natural biologically active compounds contained in medicinal plant raw materials.

Our research results reveal that in all cases, the total content of phenolic compounds shows a statistically significant correlation with RSA, similar to what was indicated in another study [52]. To ensure full recovery, quantitative levels and compositional profiles of the extracted compounds were analyzed in extracts with DMSO solvent and compared to the methanolic extracts. The exchange of solvent, which allowed medicinal plant extracts to be used for cell viability assays, did not compromise total antioxidant activity; in fact, solvent exchange concentrated the medicinal plant extracts by nearly twice. The highest RSA results were determined in *J. nigra* L. pericarp and fruit extract samples, collected during the ripening vegetation stage, *C. angustifolium* L. aerial part sample, collected during the massive flowering stage, and *Q. robur* L. fruit sample, collected during the ripening stage. These results highlight that during these stages, both plants and trees undergo significant metabolic changes, leading to an increase in phenolic compounds, flavonoids, and other antioxidants. These results also align with other studies, highlighting the increase in phenolic content throughout the growth and ripening stages of various fruits and highlighting the dynamic nature of phenolic compound accumulation. One of the reasons for such an increase is that these compounds serve as protective agents against oxidative stress caused by rapid cell division, respiration, and environmental stressors [53].

The combined use of HPLC UV-VIS and HPLC-ED systems demonstrates the value of using complementary analytical techniques when profiling phenolic compounds in medicinal plants [54,55]. HPLC UV-VIS provided a broad detection range, capturing compounds with UV-absorbing chromophores, while HPLC-ED resulted in enhanced sensitivity, particularly for redox-active compounds. Importantly, the use of multiple electrode potentials (300, 500, and 700 mV) in HPLC-ED allowed for the selective detection of phenolic compounds based on their redox behavior. Different compounds respond to different oxidative potentials depending on their electrochemical structure. Additionally, differences in electron transfer kinetics (reaction speed) may influence signal intensity and retention, adding another layer of compound differentiation. This electrochemical diversity highlights the added value of using HPLC-ED alongside HPLC UV-VIS when investigating the redox-related bioactivity of medicinal plant extracts [56]. Previous reports have emphasized the usage of reversed-phase HPLC systems for the early elution and detection of large ellagitannins such as oenothein B [5], and our findings confirm its predominance across multiple extraction conditions. Alongside oenothein B, other compounds such as 3,4-dihydroxybenzoic acid, gallic acid, and ellagic acid were the polyphenolic compounds within the tested specimens. Our findings align with the earlier literature describing their antioxidant potential, yet their biological effects may extend beyond antioxidation. For example, previous studies have demonstrated that 3,4-dihydroxybenzoic acid is linked as an apoptosis-inducing compound in cancer cell models. 3,4-dihydroxybenzoic acid has been shown to inhibit human gastric adenocarcinoma proliferation in a time- and dose-dependent manner [57]. Both gallic and ellagic acids have been extensively studied for their cancer-associated activities across multiple pathways. A recent review highlights gallic acid’s antioxidant, anti-inflammatory, and anticancer potential across diverse cell lines, while also acknowledging its ability to act as a pro-oxidant under certain conditions, leading to apoptosis [58]. Additionally, ellagic acid has been reviewed for its inhibitory effects on cancer cell line proliferation and induction of apoptosis in gastrointestinal and prostate cancer cell lines [59]. These mechanistic insights suggest that the phenolic composition revealed by HPLC UV-VIS and HPLC-ED does not merely describe chemical diversity but may have implications for the therapeutic potential of these medicinal plants, particularly in redox-mediated disease models. Our statistical analysis further supported these findings by showing that polyphenolic acids, which are generally more polar compounds than flavonoids, exhibited the strongest correlations with anticancer activity across the tested conditions. These results are linked to all tested medicinal plant extracts, with the exception of Canadian goldenrod. This plant species does not contain ellagic acid or its derivatives, and its chemical profile and observed strong anticancer activity appears to be more closely associated with high flavonoid levels and/or alkaloids, as reported in previous studies [60].

In this study, we determined 4T1 (a breast cancer cell line derived from mammary gland tissue of a mouse), A549 (adenocarcinoma human alveolar basal epithelial cell line), Caki-1 (a human kidney carcinoma cell line), HCT116 (a human colon cancer cell line), and MCF7 (a human breast cancer cell line) cancer cell line viability after exposing them to different concentrations of antioxidants, found in various medicinal plant raw materials. Since the MTS assay reflects metabolic activity rather than direct cell death, the 24 h viability measurements should primarily be interpreted as indicators of antiproliferative activity. At this early point, cells are in the exponential growth phase following plating, and reduced metabolic activity likely reflects inhibition of cell proliferation rather than cytotoxicity. Prolonged incubation times (48–72 h), in contrast, may better represent cumulative cytotoxic effects. Half-maximal inhibitory concentrations (IC_50_) were calculated to evaluate the efficiency of inhibiting 50% of cancer cell line growth. To ensure consistency across incubation timepoints, different cell numbers were seeded for each assay (8000 cells per well for 24 h, 4000 for 48 h, 2000 for 72 h), maintaining comparable cell densities at the time of measurement. This approach ensured that observed variants in IC_50_ values reflected incubation time-dependent effects of the extracts rather than differences in initial cell concentration. The lowest IC_50_ value, showcasing the highest anticancer activity against the 4T1 cell line after 24–72 h of inhibition, was determined using Canadian goldenrod, blackcurrant, and fireweed extract samples. The A549 cancer cell line reacted differently, where the lowest IC_50_ values (highest anticancer activity) were determined in common walnut and pedunculate oak samples. The highest anticancer activity against the Caki-1 cancer cell line was demonstrated by common walnut and black walnut pericarp and fruit sample extracts. Other studies also highlight black walnut’s potential in cancer therapy, demonstrating significant anticancer effects against A549 cell lines [61]. Both HCT116 and MCF7 cancer cell lines were aggressive, and not all medicinal plant extracts inhibited their growth. Still, black walnut, Canadian goldenrod, and fireweed demonstrated the ability to inhibit cell line growth effectively compared to other plant extract samples. Our previous research has shown that fireweed extracts inhibit MCF7 and MDA-MB-468 cell line growth, which is primarily attributed to ellagic acid dimer oenothein B [5]. Other studies also demonstrated that other medicinal plants, like *Artemisia absinthium* L. and *Artemisia vulgaris* L., have considerable anticancer activity against MCF7 cell lines, highlighting that only specific samples (obtained during the massive flowering vegetation stage) have significant inhibition effects [62]. Cytotoxic activity against the HEK-293 healthy cell line was not observed when exposing this human embryonic kidney healthy cell line to various medicinal plant extracts and their biologically active compounds. Specifically, cell viability remained unchanged (close to 100% across all tested concentrations), preventing the determination of IC_50_ values. This suggests a potential selectiveness of extracts toward healthy cell lines, as anticancer activity was only observed in malignant cell lines.

The anticancer activity assays demonstrated that the relationship between extract composition and IC_50_ values were cell line-specific and incubation-time dependent. At 24 h, only limited correlations were detected between phytochemical measurements and IC_50_ values, none of which survived Bonferroni correction. After 48 h incubation, stronger patterns emerged, with exploratory negative correlations observed between 4T1 IC_50_ values and total content of phenolic compounds, radical scavenging activity, and total peak area determined with HPLC UV-VIS and HPLC-ED analyses. These results suggest that phenolic-rich and antioxidant-active extracts may contribute to increased anticancer activity in certain context [46,63], but results should be interpreted cautiously given the lack of Bonferroni significance. In contrast, the MCF7 cell line showed opposite patterns, with positive correlations between IC_50_ and phytochemical measures at earlier incubation, indicating reduced anticancer activity in polyphenolic-rich extracts for this estrogen receptor-positive cell type. Such findings align with previous reports that some plant polyphenols can act as phytoestrogens, potentially promoting estrogen-dependent cell survival pathways [64,65]. At 72 h incubation, most correlations diminished, indicating a stabilization of phytochemical-driven anticancer effects with prolonged exposure.

The IC_50_ kinetics indicated two distinct patterns of anticancer activity among tested medicinal plant extracts. In most cases, IC_50_ values decreased progressively from 24 h to 72 h, demonstrating a time-dependent increase in potency, as observed for pedunculate oak (refer to Figure 3 and Figure 6) and, to some extent, black and common walnut (refer to Figure 3). In contrast, Canadian goldenrod and fireweed in most cases exhibited strong anticancer activity already at 24 h incubation, with IC_50_ values remaining relatively stable at later incubation timepoints (refer to Figure 3, Figure 6 and Figure 7). Although this rapid and sustained anticancer activity was not seen across all cancer cell lines, it was observed in the majority of cases for these medicinal plant extracts, suggesting that they act more quickly than the other tested plants. Lastly, black walnut maintained a consistently potent profile across all incubation times (refer to Figure 5, Figure 6 and Figure 7). These findings highlight that while some medicinal plant extracts require prolonged exposure to achieve maximal activity, others show immediate and stable anticancer effects, underlying the diversity in temporal dynamics of plants’ accumulating anticancer compounds.

More interesting findings were obtained from inter-cell comparisons. Bonferroni corrected analysis revealed strong correlation between IC_50_ values of different cancer cell lines, specifically between 4T1 and HCT116 cells at 24 h incubation and between 4T1 and A549 cell lines at 48 h incubation. These results indicate shared patterns of sensitivity across certain cancer cell lines and may point to conserved mechanisms of action for specific medicinal plant extracts. The overall decrease in IC_50_ values with prolonged incubation supports a time-dependent increase in anticancer activity, although the magnitude and direction of effects varied by different cancer cell lines. Collectively, these findings highlight that both phytochemical composition and incubation period influence anticancer activity responses, but that the most statistically significant outcomes reflect cross-cell line similarities rather than direct composition activity. This highlights the need for further mechanistic studies to clarify the drivers of sensitivity patterns and to validate the preliminary phytochemical correlations observed in this study.

Although the tested medicinal plant extracts demonstrated strong antioxidant properties, their effect on cancer cell viability did not always correspond with antioxidant activity levels. In several cases, extract rich in antioxidants also induced a reduction in cell viability, which contrasts with the literature reporting that antioxidants protect mitochondria by neutralizing ROS that can damage cells and active caspase-like enzymes responsible for apoptosis [66,67]. This suggests that the observed cytotoxic effects cannot be attributed solely to antioxidant activity. Instead, the impact on cell viability is likely influenced by the combined effects of multiple phytochemical compounds within the medicinal plant extracts. Further studies are required to clarify the specific interactions and mechanisms responsible for these effects.

The associations between polyphenol levels and anticancer activity observed in this study are based on statistical correlations and do not establish direct cause and effect relationships. Although oxidative stress modulation and apoptosis induction are plausible mechanisms supported by the prior literature, these were not experimentally verified here. Furthermore, the bioavailability and metabolism of polyphenolic compounds in vivo may affect their therapeutic potential, and these aspects warrant further investigation. It should be acknowledged that with the analytical methods used, the presence of compounds unrelated to antioxidant activity cannot be entirely excluded.

Limitations of the present study should be acknowledged. Although the use of crude extracts reflects the natural complexity of plant-derived bioactive mixtures, it does not allow the identification of specific molecular contributions to the observed effects. In addition, only in vitro assays were performed, and the anticancer activity observed in cell lines may not fully translate to in vivo biological systems due to differences in metabolism, bioavailability, and compound stability. Furthermore, IC_50_ values were expressed in rutin equivalents to ensure analytical consistency across antioxidant and cytotoxicity data, which may limit direct comparison with studies reporting values per mass of extract. Future research should include compound isolation and mechanistic studies, as well as in vivo validation, to more precisely define the active compounds and confirm therapeutic relevance.

## 5. Conclusions

Our findings demonstrate the anticancer activity potential of selected medicinal plant extracts against various cancer cell lines while demonstrating no cytotoxicity towards healthy human cells. Antioxidant capacity is closely linked with anticancer activity in medicinal plant extracts predominantly containing ellagic acid and its derivatives, supporting the hypothesis that oxidative stress modulation contributes to anticancer activity. Statistical analysis revealed that polyphenolic acids, rather than flavonoids, showed strong correlations with anticancer activity, suggesting that these compounds are the key contributors to the observed activity. Different plant species showed selective efficacy across cancer cell lines, emphasizing that both phytochemical composition and cellular context are critical. Moreover, kinetic analyses revealed that some medicinal plant extracts, such as Canadian goldenrod and fireweed, showed rapid and sustained anticancer activity within the first 24 h, whereas others, like pedunculate oak, required longer incubation to reach maximal effects, highlighting variability in temporal dynamics of anticancer activity responses. The observed time-dependent pattern suggests a mechanism that may involve gradual ROS accumulation, ultimately leading to possible mitochondrial stress and caspase-dependent apoptosis. Future research should focus on in vivo validation of these findings and mechanistic studies to elucidate the precise pathways through which these compounds exert their anticancer effects. Investigating potential synergistic interactions among polyphenols and their bioavailability in physiological conditions will be essential for advancing their therapeutic applications.

## Figures and Tables

**Table 1:** Medicinal plant samples tested in this study.

No	Plant Name	Collection Date	Raw Material	Vegetation Stage
1	Stinging nettle (*U. dioica* L.)	5 June 2022	Aerial part (*Herba*)	Flower bud development
2	Common walnut (*J. regia* L.)	30 September 2022	Pericarp	Ripening
3		22 July 2022	Leaves	Ripening
4		25 July 2022	Bark	Ripening
5	Black walnut (*J. nigra* L.)	6 October 2022	Pericarp	Ripening
6		20 October 2022	Fruit	Ripening
7	Canadian goldenrod (*S. canadensis* L.)	10 August 2022	Aerial part (*Herba*)	Beginning of the flowering
8	European goldenrod (*S. virgaurea* L.)	17 August 2022	Aerial part (*Herba*)	Massive flowering
9	Pedunculate oak (*Q. robur* L.)	21 June 2022	Leaves	Ripening
10		10 September 2022	Fruit	Ripening
11		6 June 2022	Bark	Ripening
12	European dewberry (*R. caesius* L.)	30 June 2022	Leaves	Massive flowering
13	Wild strawberry (*F. vesca* L.)	30 June 2022	Leaves	Massive flowering
14	Common comfrey (*S. officinale* L.)	30 June 2022	Roots	The end of vegetation
15	Blackcurrant (*R. nigrum* L.)	5 June 2022	Leaves	Ripening
16	Raspberry (*R. idaeus* L.)	30 June 2022	Leaves	Intensive growth
17	Fireweed (*C.angustifolium* L.)	13 June 2022	Aerial part (*Herba*)	Intensive growth
18		20 June 2022	Aerial part (*Herba*)	Flower bud development
19		28 June 2022	Aerial part (*Herba*)	Beginning of the flowering
20		2 July 2022	Aerial part (*Herba*)	Massive flowering
21		14 July 2022	Aerial part (*Herba*)	The end of the flowering

**Table 2:** Quantitative identification of phenolic compounds in 75% (*v*/*v*) methanol in water extracts using HPLC UV-VIS and HPLC-ED analysis, expressed in RE mg/g (n = 3, RSD < 5%).

No	Medicinal Plant Name and Part	Compound	HPLC UV-VIS		HPLC-ED	
			C_RE_, mg/g *	Total Peak Area, mV **	C_RE_, mg/g *	Total Peak Area, µCoulombs ***
1	Stinging nettle (*U. dioica* L.). Aerial part (*Herba*)	3,4-dihydroxybenzoic acid	0.03 ± 0.001	5155.5 ± 54.7	0.12 ± 0.004	2692.0 ± 56.9
		Chlorogenic acid	0.21 ± 0.01		0.08 ± 0.003	
2	Common walnut (*J. regia* L.). Pericarp	Gallic acid	0.05 ± 0.001	3453.2 ± 79.7	0.09 ± 0.004	1052.8 ± 34.7
		3,4-dihydroxybenzoic acid	0.03 ± 0.001		0.08 ± 0.003	
		Chlorogenic acid	0.07 ± 0.002		0.08 ± 0.003	
		Ferulic acid	0.03 ± 0.001		0.12 ± 0.005	
		Rutin	0.03 ± 0.001		0.19 ± 0.008	
3	Common walnut (*J. regia* L.). Leaves	Gallic acid	0.03 ± 0.001	6385.1 ± 58.9	0.15 ± 0.006	2268.8 ± 88.6
		3,4-dihydroxybenzoic acid	0.08 ± 0.003		0.16 ± 0.007	
		Vanillic acid	0.03 ± 0.001		0.08 ± 0.003	
		2-hydroxycinammic acid	0.07 ± 0.003		0.23 ± 0.01	
		Ellagic acid	0.08 ± 0.003		0.08 ± 0.001	
4	Common walnut (*J. regia* L.). Bark	Gallic acid	0.03 ± 0.001	5370.7 ± 89.8	0.37 ± 0.015	3008.6 ± 120.7
		3,4-dihydroxybenzoic acid	0.03 ± 0.001		0.12 ± 0.005	
		Vanillic acid	0.04 ± 0.001		0.16 ± 0.007	
		Ferulic acid	0.03 ± 0.001		0.10 ± 0.003	
5	Black walnut (*J. nigra* L.). Pericarp	Gallic acid	0.18 ± 0.007	18,726.4 ± 178.4	0.11 ± 0.004	7473.6 ± 241.8
		3,4-dihydroxybenzoic acid	0.12 ± 0.002		0.09 ± 0.003	
		Caffeic acid	0.44 ± 0.012		0.12 ± 0.005	
		Ferulic acid	0.24 ± 0.01		0.32 ± 0.01	
		trans-sinapic acid	0.22 ± 0.01		0.36 ± 0.01	
6	Black walnut (*J. nigra* L.). Fruit	Gallic acid	0.06 ± 0.002	3979.3 ± 44.8	0.08 ± 0.001	2381.9 ± 59.3
		3,4-dihydroxybenzoic acid	0.04 ± 0.001		0.08 ± 0.001	
		Caffeic acid	0.08 ± 0.002		0.12 ± 0.004	
		Ferulic acid	0.10 ± 0.002		0.12 ± 0.005	
		trans-sinapic acid	0.08 ± 0.002		0.14 ± 0.006	
7	Canadian goldenrod (*S. canadensis* L.). Aerial part (*Herba*)	3,4-dihydroxybenzoic acid	0.03 ± 0.001	22,017.6 ± 218.7	0.09 ± 0.004	6102.6 ± 183.6
		Vanillic acid	0.18 ± 0.008		0.15 ± 0.006	
		2-hydroxycinammic acid	0.04 ± 0.001		0.11 ± 0.004	
		Rutin	0.04 ± 0.001		0.10 ± 0.004	
		trans-cinnamic acid	0.77 ± 0.02		-	
8	European goldenrod (*S. virgaurea* L.). Aerial part (*Herba*)	Chlorogenic acid	0.19 ± 0.01	14,739.5 ± 118.2	0.08 ± 0.001	2983.4 ± 114.8
		Ferulic acid	0.06 ± 0.001		0.09 ± 0.002	
		Salicylic acid	0.05 ± 0.002		0.08 ± 0.003	
		Rutin	0.79 ± 0.03		0.78 ± 0.02	
9	Pedunculate oak (*Q. robur* L.). Leaves	Gallic acid	0.20 ± 0.009	6367.3 ± 58.9	0.39 ± 0.01	3991.5 ± 148.7
		Caffeic acid	0.03 ± 0.001		0.09 ± 0.002	
		Ellagic acid	0.18 ± 0.008		0.11 ± 0.004	
10	Pedunculate oak (*Q. robur* L.). Fruit	Gallic acid	0.17 ± 0.002	8201.3 ± 108.9	0.10 ± 0.003	6958.8 ± 241.9
		3,4-dihydroxybenzoic acid	0.04 ± 0.001		0.14 ± 0.006	
		Chlorogenic acid	0.03 ± 0.001		0.21 ± 0.01	
		Ferulic acid	0.09 ± 0.003		0.12 ± 0.005	
		2-hydroxycinammic acid	0.06 ± 0.002		0.10 ± 0.004	
		trans-cinnamic acid	0.10 ± 0.004		-	
		Ellagic acid	0.35 ± 0.01		0.44 ± 0.02	
11	Pedunculate oak (*Q. robur* L.). Bark	Gallic acid	0.06 ± 0.001	3745.2 ± 57.8	0.85 ± 0.03	2863.6 ± 112.5
		2-hydroxycinammic acid	0.04 ± 0.001		-	
		Ellagic acid	0.20 ± 0.009		0.11 ± 0.004	
12	European dewberry (*R. caesius* L.). Leaves	Gallic acid	0.07 ± 0.003	14,023.4 ± 55.6	0.09 ± 0.002	7712.3 ± 249.5
		3,4-dihydroxybenzoic acid	0.04 ± 0.001		0.31 ± 0.01	
		2-hydroxycinammic acid	0.06 ± 0.001		0.14 ± 0.004	
		trans-cinnamic acid	0.22 ± 0.009		-	
13	Wild strawberry (*F. vesca* L.). Leaves	trans-p-coumaric acid	0.42 ± 0.02	15,863.1 ± 115.8	0.47 ± 0.02	8999.5 ± 238.6
		2-hydroxycinammic acid	0.11 ± 0.004		0.09 ± 0.002	
		Ellagic acid	0.32 ± 0.01		1.11 ± 0.03	
14	Common comfrey (*S. officinale* L.). Roots	3,4-dihydroxybenzoic acid	0.03 ± 0.001	3581.1 ± 22.8	0.11 ± 0.001	2138.6 ± 86.9
15	Blackcurrant (*R. nigrum* L.). Leaves	Gallic acid	0.03 ± 0.001	6906.2 ± 69.4	0.09 ± 0.001	3527.0 ± 127.6
		3,4-dihydroxybenzoic acid	0.06 ± 0.002		0.10 ± 0.003	
		Vanillic acid	0.06 ± 0.002		0.08 ± 0.001	
		Caffeic acid	0.03 ± 0.001		0.08 ± 0.002	
16	Raspberry (*R. idaeus* L.). Leaves	3,4-dihydroxybenzoic acid	0.04 ± 0.001	11,668.8 ± 241.8	0.38 ± 0.01	5556.35 ± 187.6
		trans-p-coumaric acid	0.03 ± 0.001		0.14 ± 0.006	
		trans-cinnamic acid	0.25 ± 0.01		-	
17	Fireweed (*C. angustifolium* L.). Aerial part (*Herba*)	Gallic acid	0.12 ± 0.005	24,617.3 ± 254.2	0.36 ± 0.01	9789.5 ± 358.4
		Oenothein B	1.29 ± 0.04		3.94 ± 0.18	
		3,4-dihydroxybenzoic acid	0.43 ± 0.02		0.10 ± 0.003	
		Ellagic acid	0.08 ± 0.01		0.20 ± 0.01	
18	Fireweed (*C. angustifolium* L.). Aerial part (*Herba*)	Gallic acid	0.03 ± 0.001	23,545.9 ± 199.8	0.68 ± 0.03	14,456.4 ± 542.8
		Oenothein B	1.16 ± 0.04		5.54 ± 0.21	
		3,4-dihydroxybenzoic acid	0.30 ± 0.01		0.10 ± 0.004	
		trans-p-coumaric acid	0.03 ± 0.001		0.17 ± 0.007	
		Ferulic acid	0.04 ± 0.001		0.11 ± 0.004	
		trans-sinapic acid	0.03 ± 0.001		0.12 ± 0.004	
		2-hydroxycinammic acid	0.11 ± 0.002		0.19 ± 0.009	
		Ellagic acid	0.11 ± 0.004		0.24 ± 0.01	
19	Fireweed (*C. angustifolium* L.). Aerial part (*Herba*)	Gallic acid	0.11 ± 0.003	28,101.1 ± 395.7	0.29 ± 0.008	10,354.0 ± 213.6
		Oenothein B	1.40 ± 0.06		3.92 ± 0.18	
		3,4-dihydroxybenzoic acid	0.38 ± 0.012		0.10 ± 0.003	
		Ferulic acid	0.03 ± 0.001		0.09 ± 0.004	
		Ellagic acid	0.08 ± 0.003		0.23 ± 0.011	
20	Fireweed (*C. angustifolium* L.). Aerial part (*Herba*)	Gallic acid	0.05 ± 0.002	17,783.2 ± 248.9	0.20 ± 0.008	11,219.1 ± 413.9
		Oenothein B	0.96 ± 0.01		4.18 ± 0.16	
		3,4-dihydroxybenzoic acid	0.20 ± 0.009		0.09 ± 0.003	
		Ferulic acid	0.03 ± 0.001		0.10 ± 0.003	
		2-hydroxycinammic acid	0.04 ± 0.001		0.16 ± 0.008	
		Ellagic acid	0.04 ± 0.001		0.24 ± 0.01	
21	Fireweed (*C. angustifolium* L.). Aerial part (*Herba*)	Gallic acid	0.11 ± 0.04	22,338.4 ± 269.9	0.23 ± 0.01	9970.7 ± 397.6
		Oenothein B	1.20 ± 0.04		4.17 ± 0.19	
		3,4-dihydroxybenzoic acid	0.27 ± 0.008		0.10 ± 0.004	
		Ellagic acid	0.06 ± 0.002		0.17 ± 0.007	

For detailed information about the sample vegetation stage, see Table 1. * Concentration expressed in rutin equivalents (RE) in mg/g. ** Total area of all registered peaks in millivolts (mV). The total peak area represents the summed chromatographic signal of all detected phenolic compounds, serving as an indicator of total phenolic abundance. *** Total area of all registered peaks in µCoulombs.

**Table 3:** Quantitative identification of phenolic compounds in 5% (*v*/*v*) DMSO in water extracts using HPLC UV-VIS and HPLC-ED analysis, expressed in RE mg/g (n = 3, RSD < 5%).

No	Medicinal Plant Name and Part	Compound	HPLC UV-VIS		HPLC-ED	
			C_RE_, mg/g *	Total Peak Area, mV **	C_RE_, mg/g *	Total Peak Area, µCoulombs ***
1	Stinging nettle (*U. dioica* L.). Aerial part (*Herba*)	3,4-dihydroxybenzoic acid	0.04 ± 0.001	5697.7 ± 121.4	0.11 ± 0.004	1424.9 ± 54.8
		Chlorogenic acid	0.22 ± 0.01		0.37 ± 0.01	
		Caffeic acid	0.03 ± 0.001		-	
2	Common walnut (*J. regia* L.). Pericarp	Gallic acid	0.04 ± 0.001	3639.2 ± 88.4	0.22 ± 0.01	2056.6 ± 63.7
		3,4-dihydroxybenzoic acid	0.03 ± 0.001		0.25 ± 0.01	
		Chlorogenic acid	0.07 ± 0.002		0.10 ± 0.004	
		Ferulic acid	0.04 ± 0.001		0.34 ± 0.01	
		Rutin	0.03 ± 0.001		0.11 ± 0.003	
3	Common walnut (*J. regia* L.). Leaves	Gallic acid	0.03 ± 0.001	5629.9 ± 112.5	0.22 ± 0.01	5234.2 ± 169.7
		3,4-dihydroxybenzoic acid	0.04 ± 0.001		0.25 ± 0.01	
		Vanillic acid	0.05 ± 0.001		0.10 ± 0.004	
		2-hydroxycinammic acid	0.06 ± 0.002		0.34 ± 0.01	
		Ellagic acid	0.16 ± 0.003		0.11 ± 0.003	
4	Common walnut (*J. regia* L.). Bark	Gallic acid	0.03 ± 0.001	3495.1 ± 86.7	0.67 ± 0.03	6701.4 ± 239.7
		3,4-dihydroxybenzoic acid	0.03 ± 0.001		0.09 ± 0.002	
		Vanillic acid	0.04 ± 0.001		0.16 ± 0.007	
		Ferulic acid	0.03 ± 0.001		0.44 ± 0.02	
5	Black walnut (*J. nigra* L.). Pericarp	Gallic acid	0.29 ± 0.01	15,526.3 ± 224.6	0.12 ± 0.004	10,489.1 ± 351.8
		3,4-dihydroxybenzoic acid	0.15 ± 0.004		0.10 ± 0.003	
		Caffeic acid	0.43 ± 0.01		0.15 ± 0.006	
		Ferulic acid	0.05 ± 0.001		0.25 ± 0.01	
		trans-sinapic acid	0.07 ± 0.002		0.36 ± 0.01	
		trans-cinnamic acid	0.27 ± 0.012		-	
6	Black walnut (*J. nigra* L.). Fruit	Gallic acid	0.03 ± 0.001	3331.0 ± 105.8	0.09 ± 0.003	5001.7 ± 198.6
		3,4-dihydroxybenzoic acid	0.03 ± 0.001		0.09 ± 0.004	
		Caffeic acid	0.06 ± 0.002		0.19 ± 0.008	
		Ferulic acid	0.07 ± 0.003		0.09 ± 0.003	
		trans-sinapic acid	0.06 ± 0.002		0.17 ± 0.007	
		trans-cinnamic acid	0.14 ± 0.05		-	
7	Canadian goldenrod (*S. canadensis* L.). Aerial part (*Herba*)	3,4-dihydroxybenzoic acid	0.03 ± 0.001	40,509.1 ± 458.8	0.13 ± 0.005	19,574.8 ± 589.6
		Vanillic acid	0.43 ± 0.01		0.15 ± 0.006	
		2-hydroxycinammic acid	0.08 ± 0.03		3.51 ± 0.15	
		Rutin	0.11 ± 0.03		0.11 ± 0.001	
		trans-cinnamic acid	1.40 ± 0.05		0.15 ± 0.006	
8	European goldenrod (*S. virgaurea* L.). Aerial part (*Herba*)	Chlorogenic acid	0.12 ± 0.004	9250.8 ± 115.8	0.09 ± 0.001	7126.5 ± 234.7
		2-hydroxycinammic acid	0.03 ± 0.001		0.13 ± 0.005	
		Rutin	0.05 ± 0.001		0.20 ± 0.008	
9	Pedunculate oak (*Q. robur* L.). Leaves	Gallic acid	0.25 ± 0.01	13,425.9 ± 182.6	0.46 ± 0.02	8125.2 ± 203.6
		Caffeic acid	0.03 ± 0.001		0.10 ± 0.003	
		Ellagic acid	0.05 ± 0.001		0.13 ± 0.005	
10	Pedunculate oak (*Q. robur* L.). Fruit	Gallic acid	0.17 ± 0.006	23,021.3 ± 259.3	0.14 ± 0.006	9266.2 ± 396.4
		3,4-dihydroxybenzoic acid	0.04 ± 0.001		0.15 ± 0.006	
		Chlorogenic acid	0.03 ± 0.001		0.34 ± 0.01	
		Ferulic acid	0.09 ± 0.003		0.14 ± 0.006	
		2-hydroxycinammic acid	0.06 ± 0.002		0.10 ± 0.004	
		trans-cinnamic acid	0.10 ± 0.004		-	
		Ellagic acid	0.35 ± 0.01		0.74 ± 0.03	
11	Pedunculate oak (*Q. robur* L.). Bark	Gallic acid	0.15 ± 0.006	8810.5 ± 148.9	2.51 ± 0.12	7868.8 ± 267.8
		Ellagic acid	0.29 ± 0.01		0.15 ± 0.006	
12	European dewberry (*R. caesius* L.). Leaves	Gallic acid	0.08 ± 0.003	17,983.0 ± 248.9	0.09 ± 0.001	17,376.2 ± 593.6
		3,4-dihydroxybenzoic acid	0.07 ± 0.002		0.46 ± 0.02	
		trans-cinnamic acid	0.19 ± 0.005		-	
13	Wild strawberry (*F. vesca* L.). Leaves	trans-p-coumaric acid	0.51 ± 0.02	16,822.8 ± 238.5	0.71 ± 0.03	21,042.0 ± 637.9
		2-hydroxycinammic acid	0.10 ± 0.03		0.08 ± 0.003	
		Ellagic acid	0.26 ± 0.01		1.36 ± 0.05	
14	Common comfrey (*S. officinale* L.). Roots	3,4-dihydroxybenzoic acid	0.03 ± 0.001	3101.6 ± 115.4	0.16 ± 0.007	4235.8 ± 157.2
15	Blackcurrant (*R. nigrum* L.). Leaves	Gallic acid	0.11 ± 0.003	11603.7 ± 193.7	0.09 ± 0.003	7149.9 ± 217.9
		3,4-dihydroxybenzoic acid	0.03 ± 0.001		0.16 ± 0.007	
		Vanillic acid	0.09 ± 0.003		0.09 ± 0.003	
		Caffeic acid	0.03 ± 0.001		0.09 ± 0.004	
16	Raspberry (*R. idaeus* L.). Leaves	Gallic acid	0.04 ± 0.001	12,810.1 ± 358.6	0.09 ± 0.003	8135.1 ± 364.8
		3,4-dihydroxybenzoic acid	0.04 ± 0.001		0.16 ± 0.007	
		Vanillic acid	0.04 ± 0.001		0.09 ± 0.003	
		trans-p-coumaric acid	0.03 ± 0.001		0.09 ± 0.004	
		trans-cinnamic acid	0.25 ± 0.01		-	
17	Fireweed (*C. angustifolium* L.). Aerial part (*Herba*)	Gallic acid	0.17 ± 0.005	60,522.1 ± 589.6	0.18 ± 0.008	29,890.5 ± 893.6
		Oenothein B	3.42 ± 0.12		11.90 ± 0.13	
		3,4-dihydroxybenzoic acid	1.19 ± 0.05		0.23 ± 0.01	
		Ellagic acid	0.09 ± 0.002		0.17 ± 0.004	
18	Fireweed (*C. angustifolium* L.). Aerial part (*Herba*)	Gallic acid	0.13 ± 0.004	54,315.7 ± 598.3	0.37 ± 0.012	31,719.4 ± 963.8
		Oenothein B	3.19 ± 0.11		12.01 ± 0.21	
		3,4-dihydroxybenzoic acid	1.03 ± 0.04		0.27 ± 0.01	
		Ellagic acid	0.08 ± 0.002		0.21 ± 0.01	
19	Fireweed (*C. angustifolium* L.). Aerial part (*Herba*)	Gallic acid	0.15 ± 0.006	53,762.1 ± 482.6	0.17 ± 0.008	24,974.0 ± 679.8
		Oenothein B	3.26 ± 0.12		9.77 ± 0.18	
		3,4-dihydroxybenzoic acid	0.90 ± 0.01		0.19 ± 0.009	
		Ellagic acid	0.12 ± 0.004		0.10 ± 0.004	
20	Fireweed (*C. angustifolium* L.). Aerial part (*Herba*)	Gallic acid	0.13 ± 0.002	58,664.3 ± 369.4	0.21 ± 0.01	25,122.9 ± 896.3
		Oenothein B	3.19 ± 0.13		9.80 ± 0.16	
		3,4-dihydroxybenzoic acid	0.84 ± 0.03		0.19 ± 0.006	
		2-hydroxycinammic acid	0.03 ± 0.001		0.25 ± 0.01	
		Ellagic acid	0.12 ± 0.03		0.14 ± 0.005	
21	Fireweed (*C. angustifolium* L.). Aerial part (*Herba*)	Gallic acid	0.12 ± 0.05	43,218.0 ± 449.6	0.28 ± 0.012	33,813.0 ± 967.8
		Oenothein B	2.99 ± 0.11		13.71 ± 0.19	
		3,4-dihydroxybenzoic acid	0.64 ± 0.02		0.19 ± 0.008	
		Ellagic acid	0.11 ± 0.004		0.16 ± 0.007	

For detailed information about the sample vegetation stage, see Table 1. * Concentration expressed in rutin equivalents (RE) in mg/g. ** Total area of all registered peaks in millivolts (mV). The total peak area represents the summed chromatographic signal of all detected phenolic compounds, serving as an indicator of total phenolic abundance. *** Total area of all registered peaks in µCoulombs.

**Table 4:** Half-maximal inhibitory concentrations (IC_50_) against tested cancer cell lines, expressed in RE mg/g (n = 3, RSD < 5%).

No	Plant Name	RSA, Expressed in RE mg/g *	Incubation Period	Half-Maximal Inhibitory Concentration (IC_50_), RE mg/g				
				4T1	A549	Caki-1	HCT116	MCF7
1	Stinging nettle (*U. dioica* L.)	35.6 ± 2.2	24 h	NA **	9.24 ± 0.02	13.84 ± 0.01	NA	NA
			48 h	9.88 ± 0.04	5.76 ± 0.04	8.20 ± 0.05	ND ***	NA
			72 h	5.52 ± 0.05	**0.36 ± 0.01**	5.12 ± 0.02	7.20 ± 0.02	NA
2	Common walnut (*J. regia* L.)	52.6 ± 2.1	24 h	8.56 ± 0.02	3.40 ± 0.04	**1.60 ± 0.02**	19.92 ± 0.06	7.76 ± 0.02
			48 h	2.88 ± 0.04	2.24 ± 0.02	2.00 ± 0.05	6.96 ± 0.09	6.72 ± 0.21
			72 h	2.24 ± 0.02	2.08 ± 0.04	**1.48 ± 0.05**	4.32 ± 0.02	4.92 ± 0.05
3	Common walnut (*J. regia* L.)	42.4 ± 1.0	24 h	12.44 ± 0.05	5.28 ± 0.08	5.08 ± 0.08	21.60 ± 0.08	10.28 ± 0.02
			48 h	5.44 ± 0.08	5.40 ± 0.05	3.00 ± 0.01	7.52 ± 0.01	4.72 ± 0.01
			72 h	3.12 ± 0.10	4.36 ± 0.04	2.00 ± 0.02	8.48 ± 0.08	4.56 ± 0.06
4	Common walnut (*J. regia* L.)	99.4 ± 1.9	24 h	7.40 ± 0.07	1.36 ± 0.02	5.72 ± 0.07	ND	8.60 ± 0.05
			48 h	2.20 ± 0.04	1.20 ± 0.02	3.72 ± 0.09	9.16 ± 0.08	6.12 ± 0.02
			72 h	2.52 ± 0.06	0.40 ± 0.01	2.36 ± 0.10	5.92 ± 0.11	4.48 ± 0.11
5	Black walnut (*J. nigra* L.)	206.1 ± 1.4	24 h	6.28 ± 0.02	1.65 ± 0.05	2.56 ± 0.11	**5.28 ± 0.05**	8.56 ± 0.05
			48 h	1.76 ± 0.01	1.45 ± 0.02	2.56 ± 0.15	**2.56 ± 0.02**	7.24 ± 0.01
			72 h	1.76 ± 0.02	0.52 ± 0.02	2.56 ± 0.06	2.56 ± 0.06	3.96 ± 0.06
6	Black walnut (*J. nigra* L.)		24 h	9.48 ± 0.05	1.60 ± 0.05	3.16 ± 0.05	13.08 ± 0.02	11.76 ± 0.02
		201.4 ± 2.5	48 h	2.48 ± 0.02	1.28 ± 0.01	**1.56 ± 0.01**	4.92 ± 0.07	9.04 ± 0.06
			72 h	1.72 ± 0.05	0.64 ± 0.07	1.56 ± 0.02	2.96 ± 0.08	8.44 ± 0.11
7	Canadian goldenrod (*S. canadensis* L.)	95.2 ± 2.4	24 h	**2.92 ± 0.05**	2.20 ± 0.05	3.16 ± 0.01	6.96 ± 0.01	**4.04 ± 0.01**
			48 h	1.38 ± 0.07	2.20 ± 0.06	3.60 ± 0.06	4.20 ± 0.01	**3.08 ± 0.06**
			72 h	1.44 ± 0.02	1.32 ± 0.08	2.88 ± 0.01	4.96 ± 0.02	**2.16 ± 0.01**
8	European goldenrod (*S. virgaurea* L.)	80.4 ± 1.4	24 h	6.96 ± 0.05	5.00 ± 0.02	6.52 ± 0.07	14.96 ± 0.06	ND
			48 h	5.64 ± 0.06	3.80 ± 0.08	4.40 ± 0.07	13.52 ± 0.05	ND
			72 h	2.52 ± 0.01	4.40 ± 0.02	3.40 ± 0.01	12.68 ± 0.01	ND
9	Pedunculate oak (*Q. robur* L.)	60.5 ± 1.9	24 h	5.08 ± 0.06	**1.04 ± 0.03**	4.48 ± 0.02	ND	13.80 ± 0.06
			48 h	1.56 ± 0.02	1.16 ± 0.02	2.56 ± 0.06	15.40 ± 0.02	10.76 ± 0.11
			72 h	0.84 ± 0.02	0.44 ± 0.02	1.64 ± 0.06	11.04 ± 0.06	8.32 ± 0.12
10	Pedunculate oak (*Q. robur* L.)	192.3 ± 4.6	24 h	8.52 ± 0.01	1.24 ± 0.01	3.48 ± 0.02	24.40 ± 0.12	17.72 ± 0.06
			48 h	2.48 ± 0.06	**0.92 ± 0.07**	1.76 ± 0.07	12.48 ± 0.06	14.28 ± 0.15
			72 h	1.28 ± 0.02	0.44 ± 0.07	1.76 ± 0.06	6.36 ± 0.06	13.08 ± 0.06
11	Pedunculate oak (*Q. robur* L.)	122.5 ± 1.5	24 h	6.52 ± 0.01	1.44 ± 0.08	5.28 ± 0.01	32.88 ± 0.24	15.44 ± 0.06
			48 h	2.24 ± 0.05	1.48 ± 0.05	3.84 ± 0.06	21.04 ± 0.06	12.32 ± 0.07
			72 h	1.00 ± 0.10	0.52 ± 0.07	1.84 ± 0.02	16.20 ± 0.22	9.96 ± 0.01
12	European dewberry (*R. caesius* L.)	79.3 ± 2.5	24 h	9.96 ± 0.11	7.60 ± 0.09	7.64 ± 0.06	19.84 ± 0.06	14.28 ± 0.08
			48 h	2.96 ± 0.06	2.00 ± 0.02	3.56 ± 0.02	7.16 ± 0.02	12.08 ± 0.11
			72 h	1.08 ± 0.02	0.52 ± 0.02	2.04 ± 0.06	5.64 ± 0.06	11.24 ± 0.06
13	Wild strawberry (*F. vesca* L.)	54.9 ± 1.4	24 h	14.76 ± 0.10	3.60 ± 0.06	8.16 ± 0.07	29.68 ± 0.01	ND
			48 h	3.15 ± 0.15	3.28 ± 0.03	6.16 ± 0.01	18.04 ± 0.01	ND
			72 h	1.40 ± 0.01	0.88 ± 0.02	2.12 ± 0.06	7.52 ± 0.06	ND
14	Common Comfrey (*S. officinale* L.)	15.2 ± 1.7	24 h	6.72 ± 0.02	6.00 ± 0.07	24.08 ± 0.16	ND	ND
			48 h	5.46 ± 0.05	6.20 ± 0.03	21.40 ± 0.01	ND	ND
			72 h	1.72 ± 0.06	3.76 ± 0.07	6.64 ± 0.06	ND	ND
15	Blackcurrant (*R. nigrum* L.)	42.2 ± 1.6	24 h	3.48 ± 0.11	1.60 ± 0.01	3.32 ± 0.06	ND	11.16 ± 0.02
			48 h	1.36 ± 0.05	1.88 ± 0.01	2.72 ± 0.01	ND	10.44 ± 0.07
			72 h	1.16 ± 0.01	0.52 ± 0.01	2.12 ± 0.06	ND	7.00 ± 0.01
16	Raspberry (*R. idaeus* L.)	134.2 ± 1.3	24 h	11.48 ± 0.05	3.20 ± 0.02	10.56 ± 0.06	21.20 ± 0.02	ND
			48 h	2.44 ± 0.02	2.68 ± 0.05	4.80 ± 0.02	6.52 ± 0.09	11.48 ± 0.16
			72 h	1.08 ± 0.06	1.00 ± 0.01	2.96 ± 0.10	6.16 ± 0.06	8.56 ± 0.12
17	Fireweed (*C. angustifolium* L.)	78.2 ± 0.9	24 h	12.20 ± 0.02	2.40 ± 0.02	12.36 ± 0.01	21.44 ± 0.01	21.80 ± 0.44
			48 h	1.76 ± 0.06	2.40 ± 0.06	6.48 ± 0.15	8.28 ± 0.06	13.32 ± 0.06
			72 h	0.92 ± 0.01	1.04 ± 0.01	3.24 ± 0.01	4.88 ± 0.21	11.56 ± 0.07
18	Fireweed (*C. angustifolium* L.)	134.6 ± 1.1	24 h	10.64 ± 0.13	1.80 ± 0.06	9.32 ± 0.06	11.68 ± 0.06	17.12 ± 0.11
			48 h	1.84 ± 0.01	1.64 ± 0.06	5.56 ± 0.06	6.12 ± 0.06	11.88 ± 0.07
			72 h	**0.28 ± 0.01**	0.76 ± 0.01	2.08 ± 0.09	**2.52 ± 0.05**	9.40 ± 0.06
19	Fireweed (*C. angustifolium* L.)	145.2 ± 0.5	24 h	10.04 ± 0.01	7.20 ± 0.05	10.76 ± 0.02	20.52 ± 0.06	20.96 ± 0.06
			48 h	2.28 ± 0.06	7.00 ± 0.02	6.36 ± 0.09	8.64 ± 0.09	14.32 ± 0.22
			72 h	1.76 ± 0.06	6.52 ± 0.05	3.84 ± 0.08	9.56 ± 0.05	12.60 ± 0.06
20	Fireweed (*C. angustifolium* L.)	160.8 ± 3.9	24 h	3.80 ± 0.07	2.80 ± 0.02	14.16 ± 0.06	9.88 ± 0.05	8.24 ± 0.15
			48 h	1.84 ± 0.02	2.40 ± 0.06	6.96 ± 0.15	3.92 ± 0.09	6.60 ± 0.16
			72 h	1.84 ± 0.06	1.04 ± 0.02	4.12 ± 0.01	5.48 ± 0.10	2.32 ± 0.06
21	Fireweed (*C. angustifolium* L.)	113.9 ± 2.5	24 h	11.44 ± 0.02	2.00 ± 0.05	12.56 ± 0.06	15.20 ± 0.12	15.44 ± 0.02
			48 h	**1.28 ± 0.06**	2.00 ± 0.06	6.12 ± 0.06	5.76 ± 0.05	8.28 ± 0.06
			72 h	1.12 ± 0.07	0.40 ± 0.02	2.68 ± 0.06	5.16 ± 0.01	5.28 ± 0.11

The numbers in bold indicate the lowest IC_50_ values, which represent the highest anticancer activity for each cell line tested using different extracts at different periods (24, 48 and 72 h). * Radical scavenging activity (RSA) determined in medicinal plant specimens in 5% *(v*/*v)* DMSO in water. ** NA–no compound activity was determined. *** ND—the half-maximal inhibitory concentration (IC_50_) was not determined because the used plant extract did not sufficiently inhibit (below 50%) cancer cell growth, and the regression curve could not be completed.

**Table 5:** Summary of key statistical results. Pearson correlation coefficients (r), *p*-values, and incubation periods are provided to describe associations between antioxidant-related variables, HPLC detection methods, and anticancer activity across cancer cell lines and incubation periods.

Variables	TPC *	TFC **	RSA ***	HPLC UV-VIS	HPLC-ED	IC_50_ (4T1)	IC_50_ (A549)	IC_50_ (Caki-1)	IC_50_ (HCT116)	IC_50_ (MCF7)
TPC *	1	r = 0.407 *p* = 0.007	r = 0.993 *p* < 0.001	r = 0.435 *p* = 0.004	r = 0.449 *p* = 0.003	r = −0.471, *p* = 0.031	n.s. ****	n.s.	n.s.	r = 0.449, *p* = 0.041
TFC **	dup. *****	1	r = 0.355 *p* = 0.021	r = 0.475 *p* = 0.001	r = 0.418 *p* = 0.006	n.s.	n.s.	n.s.	n.s.	n.s.
RSA ***	dup.	dup.	1	r = 0.397 *p* = 0.009	r = 0.416 *p* = 0.006	r = −0.468, *p* = 0.032	r = −0.434, *p* = 0.049	n.s.	n.s.	r = 0.488, *p* = 0.025
HPLC UV-VIS	dup.	dup.	dup.	1	r = 0.905 *p* < 0.001	r = −0.442, *p* = 0.045	n.s.	n.s.	n.s.	r = 0.521, *p* = 0.015
HPLC-ED	dup.	dup.	dup.	dup.	1	r = −0.486, *p* = 0.025	n.s.	n.s.	n.s.	r = 0.480, *p* = 0.028
						r = −0.500, *p* = 0.021				
IC_50_ (4T1)	dup.	n.s.	dup.	dup.	dup.	1	r = 0.661, *p* = 0.001	n.s.	r = 0.673, *p* < 0.001	r = −0.624, *p* = 0.003
					dup.					r = −0.537, *p* = 0.012
IC_50_ (A549)	n.s.	n.s.	dup.	n.s.	n.s.	dup.	1	r = 0.467, *p* = 0.033	n.s.	n.s.
								r = 0.602, *p* = 0.004		
IC_50_ (Caki-1)	n.s.	n.s.	n.s.	n.s.	n.s.	n.s.	dup.	1	n.s.	r = −0.464, *p* = 0.034
							dup.			
IC_50_ (HCT116)	n.s.	n.s.	n.s.	n.s.	n.s.	dup.	n.s.	n.s.	1	n.s.
IC_50_ (MCF7)	dup.	n.s.	dup.	dup.	dup.	dup.	n.s.	dup.	n.s.	1
						dup.				

* Total phenolic content (TPC). ** Total flavonoid content. *** Radical scavenging activity (RSA). **** No significant correlation observed (*p* ≥ 0.05); ***** Duplicate (repeated r and *p* values are omitted (marked as dup.) for clarity; each correlation is reported only once). Interpretations of correlation strength follow standard guidelines: very strong (|r| ≥ 0.9), strong (|r| ≥ 0.7–0.89), moderate (|r| = 0.3–0.69), and weak (|r| < 0.3). Statistical significance was defined as *p* < 0.05. In addition, Bonferroni correction (marked green) was applied for correlation analyses (α = 0.002 for 25 tests per timepoint) to reduce the risk of false positives; results not surviving correction are presented as exploratory trends (marked yellow).

**Table 6:** DMSO-based extracts demonstrating the highest estimated different cell line anticancer activity (×10^6^ cells killed per gram of dried raw plant material) across different incubation times.

No	Plant Name	Plant Part	Incubation Period	Millions of Cancer Cells Killed Per 1 g (×10^6^) of Medicinal Plant				
				4T1	A549	Caki-1	HCT116	MCF7
1	Canadian goldenrod (*S. canadensis* L.)	Aerial part (*Herba*)	24 h	12.33–15.07	-	-	-	8.91–10.89
2	Pedunculate oak (*Q. robur* L.)	Leaves		-	34.62–42.31	-	-	-
3	Common walnut (*J. regia* L.)	Pericarp				22.50–27.50		-
4	Black walnut (*J. nigra* L.)	Pericarp		-	-	-	6.82–8.33	-
5	Fireweed (*C.angustifolium* L.)	Aerial part (*Herba*)	48 h	25.31–37.81	-	-	-	-
6	Pedunculate oak (*Q. robur* L.)	Fruit		-	35.22–52.61	-	-	-
7	Black walnut (*J. nigra* L.)	Fruit		-	-	20.77–31.03	-	-
8	Black walnut (*J. nigra* L.)	Pericarp		-	-	-	12.66–18.91	-
9	Canadian goldenrod (*S. canadensis* L.)	Aerial part (*Herba*)		-	-	-	-	10.52–15.71
10	Fireweed (*C.angustifolium* L.)	Aerial part (*Herba*)	72 h	104.14–190.14	-	-	11.57–21.13	-
11	Stinging nettle (*U. dioica* L.)	Aerial part (*Herba*)		-	81.00–147.89	-	-	-
12	Common walnut (*J. regia* L.)	Pericarp		-	-	19.70–35.97	-	-
13	Canadian goldenrod (*S. canadensis* L.)	Aerial part (*Herba*)		-	-	-	-	13.50–24.65

Values represent the estimated number of cancer cells (×10^6^) killed per 1 g of dried plant material based on the IC_50_ values obtained from MTS cell viability assays. Ranges reflect minimum and maximum values observed across replicates (n = 3). Only extracts with measurable IC_50_ values were included in the calculation.

## Data Availability

The primary data summarized in this study are available on request from the corresponding author. The raw data are not publicly available due to low value when not analyzed, compared, and summarized.

## Abbreviations

The following abbreviations are used in this manuscript:

ROS	Reactive oxygen species
DNA	Deoxyribonucleic acid
DMSO	Dimethyl sulfoxide
UV-VIS	Ultraviolet–visible
HPLC-ED	High-performance liquid chromatography–electrochemical detection
HPLC	High-performance liquid chromatography
MTS	3-(4,5-dimethylthiazol-2-yl)-5-(3-carboxymethoxyphenyl)-2-(4-sulfophenyl)-2H-tetrazolium
DPPH	2,2-diphenyl-1-picrylhydrazyl
RPMI	Roswell Park Memorial Institute
DMEM	Dulbecco’s Modified Eagle Medium
RSD	Relative standard deviation
RE	Rutin equivalents
IC_50_	Half-maximal inhibitory concentration
FBS	Fetal bovine serum
RSA	Radical scavenging activity
TPC	Total phenolic content
TFC	Total flavonoid content
n.s.	No significant correlation observed (*p* ≥ 0.05)
4T1	Breast cancer cell line derived from mammary gland tissue of a mouse
A549	Adenocarcinoma human alveolar basal epithelial cell line
Caki-1	Human kidney carcinoma cell line
HCT116	Human colon cancer cell line
MCF7	Human breast cancer cell line
HEK-293	Healthy human embryonic kidney cell line
WHO	World Health Organization
KUBG	Kaunas Botanical Garden Herbarium
